# Two large reciprocal translocations characterized in the disease resistance-rich *burmannica* genetic group of *Musa acuminata*

**DOI:** 10.1093/aob/mcz078

**Published:** 2019-06-26

**Authors:** Marion Dupouy, Franc-Christophe Baurens, Paco Derouault, Catherine Hervouet, Céline Cardi, Corinne Cruaud, Benjamin Istace, Karine Labadie, Chantal Guiougou, Lyonel Toubi, Frederic Salmon, Pierre Mournet, Mathieu Rouard, Nabila Yahiaoui, Arnaud Lemainque, Guillaume Martin, Angélique D’Hont

**Affiliations:** 1 CIRAD, UMR AGAP, Montpellier, France; 2 AGAP, Université Montpellier, CIRAD, INRA, Montpellier SupAgro, Montpellier, France; 3 Genoscope, Institut de biologie François-Jacob, Commissariat à l’Energie Atomique (CEA), Université Paris-Saclay, Evry, France; 4 CIRAD, UMR AGAP, Guadeloupe, France; 5 Bioversity International, Montpellier, France

**Keywords:** *Musa acuminata*, banana, chromosomal rearrangement, reciprocal translocation, paired-end sequencing, genotyping by sequencing

## Abstract

**Background and Aims:**

Banana cultivars are derived from hybridizations involving *Musa acuminata* subspecies. The latter diverged following geographical isolation in distinct South-east Asian continental regions and islands. Observation of chromosome pairing irregularities in meiosis of hybrids between these subspecies suggested the presence of large chromosomal structural variations. The aim of this study was to characterize such rearrangements.

**Methods:**

Marker (single nucleotide polymorphism) segregation in a self-progeny of the ‘Calcutta 4’ accession and mate-pair sequencing were used to search for chromosomal rearrangements in comparison with the *M. acuminata* ssp. *malaccensis* genome reference sequence. Signature segment junctions of the revealed chromosome structures were identified and searched in whole-genome sequencing data from 123 wild and cultivated *Musa* accessions.

**Key Results:**

Two large reciprocal translocations were characterized in the seedy banana *M. acuminata* ssp. *burmannicoides* ‘Calcutta 4’ accession. One consisted of an exchange of a 240 kb distal region of chromosome 2 with a 7.2 Mb distal region of chromosome 8. The other involved an exchange of a 20.8 Mb distal region of chromosome 1 with a 11.6 Mb distal region of chromosome 9. Both translocations were found only in wild accessions belonging to the *burmannicoides/burmannica*/*siamea* subspecies. Only two of the 87 cultivars analysed displayed the 2/8 translocation, while none displayed the 1/9 translocation.

**Conclusion:**

Two large reciprocal translocations were identified that probably originated in the *burmannica* genetic group. Accurate characterization of these translocations should enhance the use of this disease resistance-rich *burmannica* group in breeding programmes.

## INTRODUCTION

Bananas (*Musa* genus) provide staple food for millions of people in Asia, Africa, Latin America and Oceania, and represent a major cash crop around the world. Most current cultivars are derived from *Musa acuminata* (A genome, 2*n* = 2*x* = 22), sometimes combined with *Musa balbisiana* (B genome, 2*n* = 2*x* = 22). *Musa acuminata* diverged following the Pliocene geographical isolation in distinct South-east Asian continental regions and islands (Daniells *et al.*, 2001; Perrier *et al.*, 2009; Janssens *et al.*, 2016; Rouard *et al.*, 2018). It has been divided into 6–9 partially interfertile subspecies (*M. a.* ssp. *burmannica*, *burmannicoides*, *siamea*, *malaccensis*, *truncata*, *errans*, *microcarpa*, *zebrina* and *banksii*) based on morphology and geographic distribution (Simmonds, 1962; Perrier *et al.*, 2009). The current domestication scenario suggests that plants were transported during human migrations, leading to hybridization between *Musa* species and subspecies (Perrier *et al.*, 2011). This resulted in the emergence of inter(sub)specific hybrids with reduced fertility (Dodds and Simmonds, 1948; Shepherd, 1999). Early farmers would then have selected parthenocarpic diploid and triploid hybrids producing edible fruits with high flesh and low seed content. These hybrids were then propagated clonally. The hundreds of current cultivars are thus products of centuries – in some cases millennia – of vegetative propagation. Only a few of them are cultivated at a large scale and one clonal cultivar group, i.e. Cavendish, accounts for >50 % of world banana production. These large-scale monocultures have favoured the emergence of diseases and extensive pesticide use (Lescot *et al.*, 2008).

Observations of chromosomal pairing during meiosis showed irregularities in the *M. acuminata* and *M. balbisiana* interspecific hybrid and in *M. acuminata* intersubspecific hybrids, with the presence of univalents and multivalents (Shepherd, 1999; Jeridi *et al.*, 2011). Within subspecies, meioses were generally found to be regular with bivalents. In addition, genetic mapping studies involving *M. acuminata* hybrids revealed substantial segregation distortion involving a few linkage groups (Fauré *et al.*, 1993; Hippolyte *et al.*, 2010; Mbanjo *et al.*, 2012; Noumbissié *et al.*, 2016). The presence of large chromosomal structural variations between species and subspecies was suggested to be responsible for these irregularities. Based on the study of pairing configurations in a limited number of *M. acuminata* intersubspecific hybrids, Shepherd (1999) proposed that six translocation groups exist, differing from a ‘Standard’ (ST) group by 1–3 translocations. These groups are only partly concordant with subspecies delimitation, with the ‘Standard’ group being the largest one and comprised *banksii*, *microcarpa* and *malaccensis* ssp. accessions.

The availability of a *M. acuminata* reference genome sequence (D’Hont *et al.*, 2012; Martin *et al.*, 2016) allowed us to implement next-generation sequencing (NGS) technologies and comparative genomics to investigate large structural variations within *Musa*. A first large reciprocal translocation between chromosomes 1 and 4 was identified as compared with the *M. acuminata* reference genome sequence obtained from the ‘DH-Pahang’ *M. acuminata* ssp. *malaccensis* accession. Among wild accessions, this 1/4 translocation was found only in some of the ssp. *malaccensis* accessions, suggesting that it emerged within this subspecies (Martin *et al.*, 2017). The 1/4 translocation was suggested to correspond to the ‘Northern Malayan’ karyotypic group proposed by Shepherd (1999), and the *M. acuminata* reference genome sequence was suggested to correspond to the ST group. Strong segregation distortion and reduced recombination were observed around translocation breakpoints in progeny from structurally heterozygous accessions. Interestingly, the ‘Northern Malayan’ structure was found to be preferentially transmitted to the progeny (Martin *et al.*, 2017).

Another large reciprocal translocation between chromosomes 1 and 3 and a large inversion on chromosome 5 were characterized in *M. balbisiana* as compared with the *M. acuminata* reference genome sequence (Baurens *et al.*, 2019). A high proportion of aneuploids was observed, involving chromosomes 1, 3 and 5, in the progeny of a tetraploid accession bearing this *M. balbisiana* chromosome structure in one copy and three copies of the standard *M. acuminata* structure. Moreover, high segregation distortion and reduced recombination were observed for these chromosomes (Baurens *et al.*, 2019).

These studies showed that large structural variations cause segregation distortion and recombination reduction or suppression in *Musa* structural hybrids, as observed in several other plants such as *Lens*, *Helianthus* and peach (Tadmor *et al.*, 1987; Quillet *et al.*, 1995; Jáuregui *et al.*, 2001). Characterizing such rearrangements is thus important to facilitate the use of genetic resources in breeding programmes, in particular to improve disease resistance.

In this study, we analysed the segregation of a self-progeny of a wild *M. acuminata* ssp. *burmannicoides* accession and detected two large reciprocal translocations compared with the *M. acuminata* ssp. *malaccensis* reference sequence. We then accurately characterized breakpoints of these structural variations through discordant mate-pair read analysis. We developed an *in silico* approach based on signature segment junctions to detect translocation breakpoints in paired-end sequences from 123 accessions representative of *M. acuminata* germplasm.

## MATERIALS AND METHODS

### Plant and sequencing material

A self-progeny of 76 individuals from the ‘Calcutta 4’ accession (PT-BA-00051, CRB Plantes Tropicale, Guadeloupe) was produced at the CIRAD research station in Guadeloupe. DNA was extracted, and sequencing libraries were constructed using a genotyping by sequencing (GBS) approach and sequenced on the GeT-PlaGe platform (https://get.genotoul.fr/) using the Illumina Hiseq3000 sequencer.

Other banana plant material was obtained either from CRB Plantes Tropicales (Guadeloupe, French West Indies, http://crb-tropicaux.com/Portail) or from the Bioversity International *Musa* Germplasm Transit Centre (Ruas *et al.*, 2017) (Supplementary data Table S1).

A total of 123 accessions were sequenced to obtain standard Illumina 2 × 150 paired-end reads using the HiSeq4000 platform at Genoscope (http://www.genoscope.cns.fr).

For 11 of these accessions, 5 kb insert mate-pair libraries were constructed and sequenced using the Illumina Hiseq2500 platform at Genoscope (http://www.genoscope.cns.fr). This data set was supplemented by Illumina 5 kb mate-pair sequencing data generated by Rouard *et al.* (2018) for three accessions (‘Banksii’, ‘Maia’Oa’ and ‘Calcutta 4’) and deposited in the National Center for Biotechnology Information (NCBI) Sequence Read Archive (SRA) under run IDs SRR7013759, SRR7013765 and SRR7013755. The assembly of the *M. acuminata* ssp. *banksii* accession ‘Banksii’ published by these authors, as well as PacBio reads related to this project (SRA ID SRR7013757), were also used.

Finally, ‘Calcutta 4’ and ‘Maia’Oa’ accessions were sequenced with a MinION nanopore sequencer from Oxford Nanopore Technologies (Jain *et al.*, 2016). As described by Belser *et al.* (2018), a 30× sub-set of the longest nanopore reads was extracted from the total read set. These reads were then assembled using the Smartdenovo assembler (Jue Ruan, https://github.com/ruanjue/smartdenovo, git-commit 3d9c22e) with –k 17, as advised by the developer for larger genomes, and –c 1 to generate a consensus sequence.

### SNP marker segregation analysis

Raw GBS sequencing data from the self-progeny were demultiplexed using GBSX version 1.2 (Herten *et al.*, 2015) and adaptors were removed with cutadapt (Martin, 2011). Variant calling was processed as described by Garsmeur *et al.* (2018), using the VcfHunter package (https://github.com/SouthGreenPlatform/VcfHunter). Mapping was performed on the *M. acuminata* reference sequence version 2 (Martin *et al.*, 2016). Sites with read depths of <10 and >1000 were excluded. Sites with a minor allele frequency of <5 % were considered homozygous. Sites with an alternate allele frequency of >15 % supported by at least three reads were considered heterozygous. Other sites were converted into missing data. single nucleotide polymorphism (SNP) markers were then filtered out based on their segregation ratio in the mapping population: a *P*-value threshold of 4e-06 was applied with a *χ*^2^ test to compare the observed marker segregation with the expected marker segregation (1:2:1 with a self-population). SNP markers with >2.5 % missing data and individuals with >1% missing data were removed from the SNP data set. Finally, 67 individuals were kept and 5281 SNP markers were selected to build the genetic map.

### Five kilobase discordant mate-pair read analysis

To refine the location of the rearrangement breakpoints and to characterize the chromosome structures of 14 accessions, large insert mate-pair reads from the 14 mate-pair sequenced accessions were aligned to the *M. acuminata* ‘DH-Pahang’ reference genome sequence (version 2) available on the Banana Genome Hub (Droc *et al.*, 2013). They were analysed to identify discordant read clusters according to the methodology described in Martin *et al.* (2017), using Scaffremodler tools (Martin *et al.*, 2016) available on the South Green platform (Southgreen Collaborators, 2016). Discordant read clusters were viewed using CIRCOS software (Krzywinski *et al.*, 2009).

### Alignment of translocation breakpoints between ‘DH-Pahang’ and ‘Calcutta 4’

To precisely compare the rearrangement breakpoints between ‘DH-Pahang’ and ‘Calcutta, the four genomic regions of the ‘DH-Pahang’ reference genome (version 2) corresponding to translocation breakpoint regions (chromosome 1 between 8.23 and 8.27 Mb, chromosome 2 between 29.26 and 29.28 Mb, chromosome 8 between 37.715 and 37.735 Mb and chromosome 9 between 11.57 and 12.61 Mb) were aligned to the ‘Calcutta 4’ preliminary assembly using blastn (BLAST+ 2.6.0; Camacho *et al.*, 2009) with an e-value threshold of 1e-20 and without masking low-complexity regions. Then comparisons between sequences were performed using Gepard (Krumsiek *et al.*, 2007) (Supplementary data Table S2).

### Signature segment junction detection

In order to characterize the chromosome structure of the 123 studied *Musa* accessions, paired reads from 123 accessions were aligned to the complete *M. acuminata* ‘DH-Pahang’ reference genome, the ‘Calcutta 4’ assembly, the ‘Maia’Oa’ assembly and the ‘Banksii’ corrected assembly using bowtie2 in very sensitive mode (see next section). Pairs properly mapped with reads on either side of a signature segment junction (SSJ; i.e. the junction between two genomic segments that is specific to one of the chromosome structures; Supplementary data Fig. S1) in a flanking window of 1000 bp around the SSJ were counted.

Local reassembly of the chromosome 8 translocation breakpoint in ‘Maia’Oa’ and ‘Banksii’ accessions

To precisely characterize the positions of the SSJs relative to the breakpoint region of chromosome 8 in two accessions representative of *zebrina* and *banksii* subspecies, long reads from ‘Maia Oa’ and ‘Banksii’ were compared with the *M. acuminata* ‘DH-Pahang’ reference chromosome 8 breakpoint region. Raw long reads from ‘Maia’Oa’ and ‘Banksii’ accessions were corrected using the correct option of Canu 1.6 (Koren *et al.*, 2017) with default parameters. Corrected reads were aligned to the *M. acuminata* ‘DH-Pahang’ reference genome version 2 using Graphmap (Sović *et al.*, 2016) with default parameters. For each accession, the longest read overlapping the two SSJs of *M. acuminata* ‘DH-Pahang’ reference chromosome 8 was kept. Selected long reads were corrected by aligning Illumina short reads from their respective accessions with bwa mem (Li and Durbin, 2009) with default parameters. Consensus between short reads was calculated with bcftools (Li, 2011).

The resulting consensus sequences were compared with the 37.718–37.732 Mb region of *M. acuminata* ‘DH-Pahang’ reference chromosome 8 using Gepard. Then we compared the ‘Maia’Oa’ consensus sequences of the chromosome 8 breakpoint with the ‘Maia’Oa’ scaffolds and the ‘Banksii’ consensus sequences of the chromosome 8 breakpoint with the ‘Banksii’ scaffolds using blastn, with an e-value threshold of 1e-20, while not masking low-complexity regions to confirm that the region has been correctly assembled. An inconsistency between the ‘Banksii’ corrected long read and its assembly was noticed, probably due to a short read assembly error. The genomic region in the original assembly was thus replaced by the new consensus sequence. Finally, the corresponding regions of the ‘Banksii’ and ‘Maia’Oa’ assembly were aligned and compared with the 1000 bp flanking window around the two *M. acuminata* ‘DH-Pahang’ reference chromosome 8 SSJs.

### Factorial analysis using SNP data

To investigate the distribution of the translocated chromosomes in *M. acuminata*, a factorial analysis was performed. Paired-end reads (2 × 150) Illumina sequencing data from the 83 *M. acuminata* diploid accession studied were used to produce a genotype call for each accession using vcfHunter tools (Garsmeur *et al.*, 2018; https://github.com/SouthGreenPlatform/VcfHunter). The process_reseq_1.0.py and VcfPreFilter.1.0.py programs were used to (1) align Illumina reads along the *M. acuminata* ‘DH-Pahang’ reference genome sequence (version 2) (Martin *et al.*, 2016) using Burrows–Wheeler Alignment version 0.7.15; (2) remove duplicates using the MarkDuplicates option of Picard Tools (version 2.7) (http://broadinstitute.github.io/picard/); (3) locally realign reads around indels using the IndelRealigner tool of the GATK software package (version 3.6) (McKenna *et al.*, 2010); (4) count all mapping bases that had a mapping quality ≥10 with the bam-readcount program (https://github.com/genome/bam-readcount); and (5) call a genotype based on the maximum likelihood of the genotype according to a binomial distribution assuming a sequencing error rate of 0.005. The variant calling file was formatted in VCF format. The VCF file was then filtered using the vcfFilter.1.0.py program with the following options: a minimal coverage by accession of 10 (–MinCov 10), a maximal coverage by accession of 119 (–MaxCov 119), a minimal relative allele frequency by accession of 0.1 (–MinFreq 0.1), a minimal absolute allele frequency by accession of 3 (–MinAl 3), no missing genotypes in a line allowed (–nMiss 0), filtering out indels (–RmType INDELS) and non-diallelic sites (–RmAlAlt 1:3:4:5). A sub-set of 3092 polymorphous SNPs dispersed along the 11 chromosomes of the reference sequence was used to calculate a dissimilarity matrix using vcf2dis.1.0.py script. The dissimilarity index between two accessions was calculated from the VCF file as the proportion of unmatching alleles between two accessions. The dissimilarity matrix was used to perform a factorial analysis using R version 3.2.4 software (http://www.r-project.org). Since the cultivated accessions originated from the wild banana genepool, the factorial analysis was performed with the 32 wild accessions. The 51 cultivated accessions were then projected along the synthetic axes.

## RESULTS

### Genetic evidence of two large reciprocal translocations in the ‘Calcutta 4’ accession

**Fig. 1.**
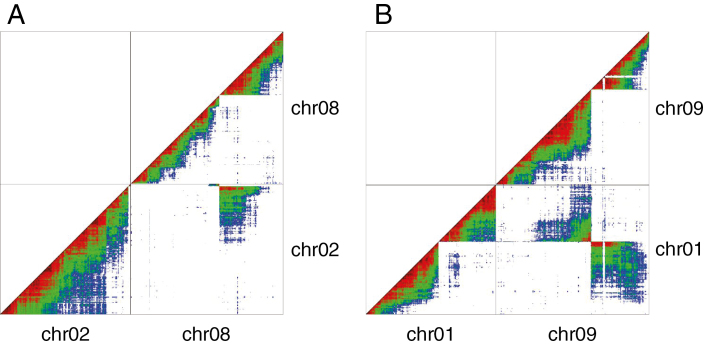
Genetic linkage between SNP markers in the ‘Calcutta 4’ self-progeny projected on *M. acuminata* reference chromosomes 2 and 8 (A) and 1 and 9 (B). Each dot represents linkage between two markers. Linkage intensity is represented by a warm–cool colour gradient from dark red (strong) to blue (weak).

A self-progeny from the ‘Calcutta 4’ accession was genotyped by sequencing. A total of 5281 markers segregating in 67 individuals with a 1:2:1 ratio were selected. Pairwise associations between ‘Calcutta 4’ markers projected on the 11 *M. acuminata* reference chromosome sequence showed, for most markers physically close on the reference sequence, strong genetic linkage, as expected (Supplementary data Fig. S2A, B). However, a complete break in genetic linkage was observed within a few chromosomes. Linkage breaks were observed on chromosome 2 at position 29.2 Mb and on chromosome 8 at position 37.7 Mb (Fig. 1A). In addition, a small cluster of markers (four markers) from the distal region of reference chromosome 2 was found in ‘Calcutta 4’ genetically linked with chromosome 8 markers, whereas a distal part of reference chromosome 8 was found to be linked with chromosome 2 in ‘Calcutta 4’.

A similar situation was observed for chromosomes 1 and 9. Linkage breaks were observed on chromosomes 1 and 9 at 8.2 and 11.4 Mb, respectively (Fig. 1B). Markers surrounding these breaks linked, in ‘Calcutta 4’, a distal region of reference chromosome 1 to reference chromosome 9 and the rest of chromosome 1 to a distal part of reference chromosome 9.

These observations suggested the presence of distinct chromosome structures in ‘Calcutta 4’ as compared with the *M. acuminata* reference sequence. These structures resulted from two reciprocal translocations, one involving chromosomes 1 and 9 and the other involving chromosomes 2 and 8. The observed linkage breaks were complete, indicating that ‘Calcutta 4’ is structurally homozygous for these translocations.

### Characterization of the translocation breakpoints

Translocation breakpoints were further characterized using 5 kb mate-pair reads from ‘Calcutta 4’ aligned to the ‘DH-Pahang’ reference sequence to search for discordant read alignments.

**Fig. 2.**
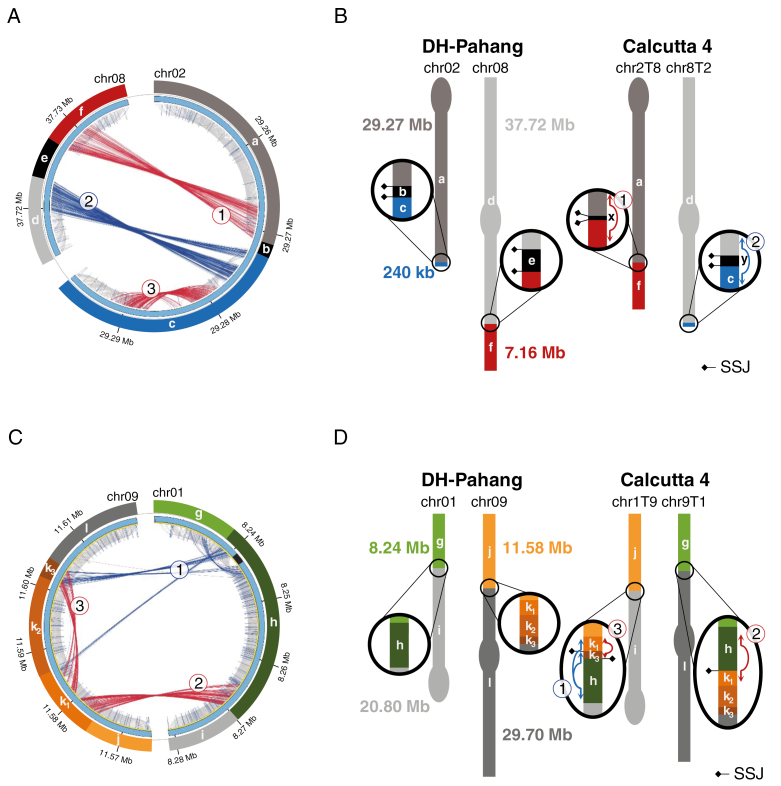
Paired-read mapping evidence for two reciprocal translocations in ‘Calcutta 4’ and schematic chromosomal diagram representations. (A and C) Circos representation of significant discordant read clusters from ‘Calcutta 4’ compared with the *M. acuminata* reference sequence assembly, with a focus on paired read clusters detected in the targeted regions of chromosomes 2 and 8, and chromosomes 1 and 9. Grey internal lines correspond to ‘Calcutta 4’ paired reads with correct orientation and insert size, while red and blue lines correspond to discordant pairs with higher insert size and reverse mapping orientation, respectively. Note: cluster 3 on segment c indicates a small deletion in ‘Calcutta 4’ outside the translocation event. (B and D) Schematic chromosome structures for ‘Calcutta 4’ chromosomes based on the paired-read mapping interpretation. Black segments are accession specific. Signature segment junctions (SSJs) are represented with diamonds.

For chromosomes 2 and 8, two clusters of discordant reads were detected in the region of chromosomes 2 and 8 and found to be genetically linked in the self-progeny of ‘Calcutta 4’ (Fig. 2A). Using Scaffremodler, these discordant read clusters were identified as resulting from the presence of a reciprocal translocation in ‘Calcutta 4’ as compared with the reference sequence (Fig. 2B). Cluster 2 linked a distal region of 240 kb (c) of chromosome 2 to 45 Mb of chromosome 8 (d), resulting in a distinct chromosome structure hereafter referred to as 8T2 (Fig. 2B). Cluster 1 linked a distal region of 7.2 Mb from chromosome 8 (f) to 29.3 Mb of chromosome 2 (a), resulting in a distinct chromosome structure hereafter referred to as 2T8 (Fig. 2B). On reference chromosomes 2 and 8, the two discordant read clusters are separated by small chromosome segments of 3 kb (e) and 1.2 kb (b), respectively, that were not covered by any read sequences in ‘Calcutta 4’, thus suggesting their absence in this accession (Fig. 2A). No concordant reads overlapped the breakpoint regions, thus indicating that ‘Calcutta 4’ is homozygous for this translocation.

For chromosomes 1 and 9, the Scaffremodler program could not automatically interpret the discordant read cluster pattern. Therefore, we manually explored discordant read clusters linking those chromosomes and found a complex pattern of interchromosomal and intrachromosomal discordant read clusters (Fig. 2C). Discordant read cluster 3 and concordant reads along the k2 segment indicated the deletion of this segment in one chromosome. Discordant read cluster 1 linked the k_1_–k_3_ segment to the h segment, resulting in k_1_–k_3_–h and k_1_–k_2_–k_3_–l chromosome fragments. Finally, cluster 2 linking the h fragment to the k_1_ fragment reduced possible combinations to reconstruct the chromosome regions, without missing any segment, to a g–h–k_1_–k_2_–k_3_–l structure for one chromosome and a j–k_1_–k_3_–h–i structure for the other. These chromosome structures corresponded to a reciprocal translocation that occurred in two distinct chromosomes. The first one, hereafter referred to as 1T9, linked a 8.24 Mb distal region of chromosome 1 (g) with 29.7 Mb of chromosome 9 (l). The second one, hereafter referred to as 9T1, linked an 11.6 Mb distal region of chromosome 9 (j), with 20.8 Mb of chromosome 1 (i) (Fig. 2D).

### Refining the translocation breakpoints in ‘Calcutta 4’

**Fig. 3.**
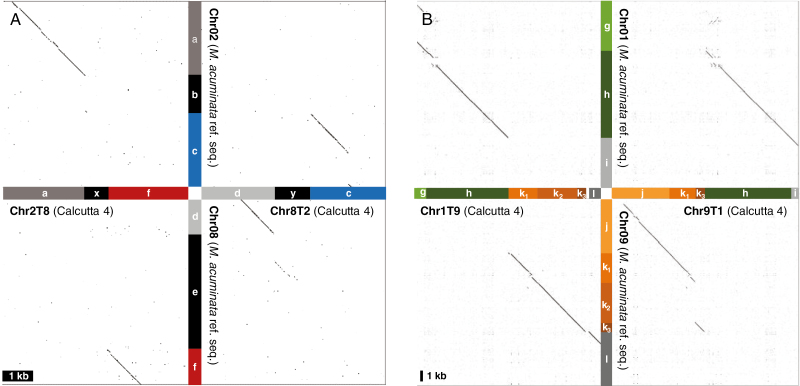
Dot-plot representation of alignments between ‘Calcutta 4’ scaffolds and the *M. acuminata* reference chromosome sequence at translocation breakpoints. (A) Translocation 2/8: ‘Calcutta 4’ for breakpoint regions of chromosomes 2T8 (scaffold utg154) and 8T2 (scaffold utg170) are represented on the horizontal axis. The corresponding regions in the *M. acuminata* reference genome are represented on the vertical axis. Each compared region is 6 kb long. A small deletion in segment c of chromosome 2 can be noticed, which corresponds to part of the 5 kb deletion observed in Fig. 2A. (B) Translocation 1/9: ‘Calcutta 4’ for breakpoint regions of chromosomes 1T9 (scaffold utg195) and 9T1 (scaffold utg94) are represented on the horizontal axis. The corresponding regions in the *M. acuminata* reference genome are represented on the vertical axis. Each compared region is 70 kb long. The colour coding is the same as in Fig. 2.

A ‘Calcutta 4’ preliminary assembly was compared with the ‘DH-Pahang’ *M. acuminata* reference sequence to identify ‘Calcutta 4’ scaffolds overlapping the translocation breakpoints. Two large scaffolds were identified for the 2/8 translocation. One scaffold (2.54 Mb long) contained the 2T8 breakpoint with a segment that aligned to reference chromosome 2 (a) and a segment that aligned to reference chromosome 8 (f) separated by a 600 bp segment (x) with no similarity to the corresponding region in reference chromosomes 2 (b) or 8 (e) (Fig. 3A). The second scaffold (3.15 Mb long) contained the 8T2 breakpoint, with a segment that aligned to reference chromosome 8 (d) and another that aligned to reference chromosome 2 (c) separated by a 1.2 kb segment (y), with no similarity to the corresponding region in reference chromosomes 2 (b) or 8 (e) (Fig. 3A).

For the 1/9 translocation, two scaffolds were also identified. One scaffold (6.9 Mb long) contained the 1T9 breakpoint, with segments that aligned to reference chromosome 1 (g and h) and segments that aligned to reference chromosome 9 (k_1_, k_2_, k_3_ and l) (Fig. 3B). The second scaffold (5.7 Mb long) contained the 9T1 breakpoint, with segments that aligned to reference chromosome 9 (j, k_1_ and k_3_) and segments that aligned to chromosome 1 (h and i).

These data validated the proposed chromosome structures based on discordant read analysis and revealed the presence, at the breakpoints, of chromosomes 2T8 and 8T2 of segments (x and y) not present in the reference sequence.

### Distribution of the two translocations in *Musa* germplasm

#### Five kilobase mate-pair discordant read analysis.

To analyse the distribution of the two translocations in *Musa* germplasm, we first used 5 kb mate-pair reads available from 13 *M. acuminata* accessions (five wild seedy accessions and eight hybrid cultivated accessions; Supplementary data Table S1). Paired reads were aligned to the ‘DH-Pahang’ reference sequence, and discordant clusters identified in ‘Calcutta 4’ as corresponding to the 2T8, 8T2, 1T9 and 9T1 chromosome structures were searched (Supplementary data Fig. S3A, B).

Among the 13 *M. acuminata* accessions, 2T8 and 8T2 chromosome structures were detected in one accession, i.e. the ‘Manang’ diploid cultivar. In this accession, concordant mate-pair reads spanning the breakpoints were also observed, revealing that this accession is structurally heterozygous, with one set of reference chromosomes 2 and 8 and one set of chromosome structures 2T8 and 8T2. All other accessions were found to be homozygous with two sets of reference chromosomes 2 and 8 (Supplementary data Fig. S3A).

No discordant clusters corresponding to chromosomes 1T9 and 9T1 were observed in the 13 accessions surveyed, and all accessions showed concordant mate-pair reads corresponding to reference chromosomes 1 and 9. These accessions were thus homozygous for reference chromosomes 1 and 9 (Supplementary data Fig. S3B).

#### Signature segment junctions searched in whole-genome sequencing (WGS) data.

To further characterize the distribution of 2T8, 8T2, 1T9 and 9T1 chromosome structures in *Musa* diversity, we used the Illumina paired-end sequence generated on 123 accessions. These accessions included 97 *M. acuminata* accessions with 32 diploid wild seedy accessions and 65 cultivated intersubspecific hybrids (51 diploids and 14 triploids), four diploid accessions from other species (two *M. balbisiana* and two *M. schizocarpa*) and 22 interspecific cultivated hybrids involving *M. acuminata* (four diploids and 18 triploids) (Supplementary data Table S1).

In the distinct chromosomal structures, distinct chromosomal segments were juxtaposed, so a junction between chromosome segments could be specific to one chromosomal structure and constitute a signature of this structure (hereafter referred to as an SSJ). Such SSJs were searched at the 2/8 and 1/9 translocation breakpoints in ‘Calcutta 4’ and in the corresponding region of the ‘DH-Pahang’ reference genome sequence.

Eight SSJs could be identified for translocation 2/8, with two per chromosome structure: a–b and b–c segment junctions for chromosome 2, d–e and e–f segment junctions for chromosome 8, a–x and x–f segment junctions for chromosome 2T8, and d–y and y–c segment junctions for chromosome 8T2 (Fig. 2; Supplementary data Table S3).

**Fig. 4.**
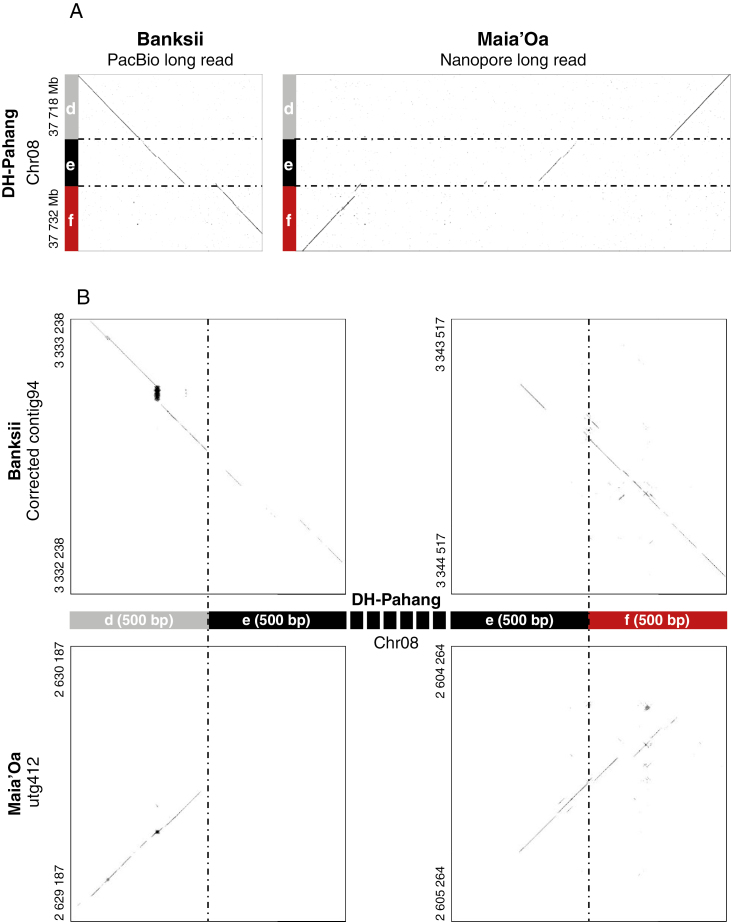
Dot-plot representation of alignments between the *M. acuminata* reference chromosome 8 and ‘Maia’Oa’ and ‘Banksii’ sequences in the region corresponding to the 2/8 translocation breakpoints. (A) Macrostructure: *M. acuminata* reference sequence for chromosome 8 (from 37 718 Mb to 37 732 Mb) is represented on the vertical axes. Homologous ‘Banksii’ PacBio long read and ‘Maia’Oa’ nanopore long reads are represented on the horizontal axis. (B) Microstructure: 500 bp regions of the *M. acuminata* reference chromosome 8 flanking the d–e and e–f segment junction is represented on the horizontal axis. Homologous regions in ‘Banksii’ (corrected contig94) and ‘Maia’Oa’ (scaffold utg412) are represented on the vertical axes. The colour coding is the same as in Fig. 2.

In a first round of analysis, for many accessions we detected SSJs of reference chromosome 2 but did not detect the signatures of reference chromosome 8. We thus compared the d–e–f breakpoint region of ‘DH-Pahang’ with the corresponding region of two additional accessions, for which long read sequences and sequence scaffolds were available (accession ‘Banksii’ from ssp. *banksii* and accession ‘Maia’Oa’ from ssp. *zebrina*). The comparison revealed small structural variations in ‘Maia’Oa’ and ‘Banksii’ compared with ‘DH-Pahang’ (Fig. 4). These structural variations, when present in the tested accession, resulted in paired-end reads that could not map on the sequence used as reference for the alignment (here ‘DH-Pahang’), which may explain why SSJs of reference chromosome 8 could not be detected in some accessions. To increase the chance of detecting SSJs corresponding to chromosome 8, we accurately identified their positions on the ‘Maia’Oa’ preliminary assembly and the ‘Banksii’ corrected assembly, and aligned paired-end data from the 123 accessions using these assemblies as additional references (Supplementary data Table S1) (see the Materials and Method for ‘Banksii’ assembly correction).

The expected SSJs were detected for the four accessions used as sequence template to align the paired reads (‘DH-Pahang’, ‘Maia’Oa’, ‘Banksii’ and ‘Calcutta 4’). SSJs of chromosome 2T8 and of chromosome 8T2 were detected as expected in ‘Calcutta 4’. SSJs of chromosome 2 were detected in ‘Pahang’, ‘Maia’Oa’ and ‘Banksii’. However, SSJs of reference chromosome 8 were detected in paired-end reads from ‘Pahang’, ‘Maia’Oa’ or ‘Banksii’ only when alignment was performed on scaffolds from the same accession. This confirmed that small structural variations within accessions can hamper proper detection of SSJs and thus that only a positive result for the presence of an SSJ can be directly interpreted with confidence. However, since ‘DH-Pahang’, ‘Banksii’ and ‘Maia’Oa’ share the same macrostructure for chromosome 8 (Fig. 4A), the detection of SSJs in any of these three accessions was hereafter interpreted as indicating the presence of the chromosome 8 structure.

Among the 123 accessions tested, only seven displayed signatures of chromosome 2T8 and chromosome 8T2. Five wild *M.* accessions (‘Long Tavoy’, ‘Pisang Prentel’, ‘Calcutta 4’, ‘Khae Phrae’ and ‘Pa Rayong’) displayed the four signatures of chromosome 2T8 and chromosome 8T2, but no SSJs of reference chromosomes 2 and 8. Moreover, two *M. acuminata* cultivated accessions displayed some signatures of chromosomes 2T8 and 8T2. Accession ‘Hom’ had four SSJs of reference chromosomes 2 and 8 and three signatures of chromosomes 2T8 and 8T2, and was thus classified as structurally heterozygous with chromosomes 2, 8, 2T8 and 8T2 structures. The ‘Manang’ accession had three SSJs for reference chromosomes 2 and 8 and two SSJs for chromosome 2T8, thus supporting its structurally heterozygous classification based on the 5 kb mate-pair discordant read analysis.

Among the remaining 90 *M. acuminata* accessions, 82 displayed the four SSJs for reference chromosomes 2 and 8 and eight accessions had at least one signature for reference chromosome 2 and one SSJ for reference chromosome 8 (Supplementary data Table S1). Two other *M. acuminata* accessions (‘Pa Patthalong’ and ‘Pisang Serun 400’) had SSJs only for reference chromosome 2.

Among the four accessions representative of other *Musa* species, one *M. schizocarpa* accession (ITC0926) had the four signatures of chromosomes 2 and 8. The other *M. schizocarpa* accession (ITC0599) and both *M. balbisiana* accessions only displayed the two SSJs of reference chromosome 2.

Finally, the 22 interspecific hybrid accessions showed 3–4 SSJs of chromosomes 2 and 8, and were thus identified as having at least one copy of reference chromosomes 2 and 8.

Regarding the 1/9 translocation, the intricate duplications at translocation breakpoints complicated the identification of SSJs specific to the four chromosome structures. We identified two SSJs for chromosome 1T9 that corresponded to k_1_–k_3_ and k_3_–h segment junctions, and one signature position for chromosome 9T1 that corresponded to the h–k_1_ segment junction (Supplementary data Table S4). No segment junctions were specific to reference chromosomes 1 and 9, so no SSJs could be used to reveal these chromosome structures.

Within the 123 accessions tested, five accessions displayed one SSJ for chromosome 1T9 (SSJ k_1_–k_3_) (‘Calcutta 4’, ‘Pisang Prentel’, ‘Long Tavoy’, ‘Khae Phrae’ and ‘Pa Rayong’). In addition, ‘Calcutta 4’ showed the SSJ of chromosome 9T1 (Supplementary data Table S1). Among them, ‘Calcutta 4’ proved to be homozygous for chromosome structures 1T9 and 9T1 based on the segregation analysis findings.

### Factorial analysis with *Musa acuminata* diploid accessions.

**Fig. 5.**
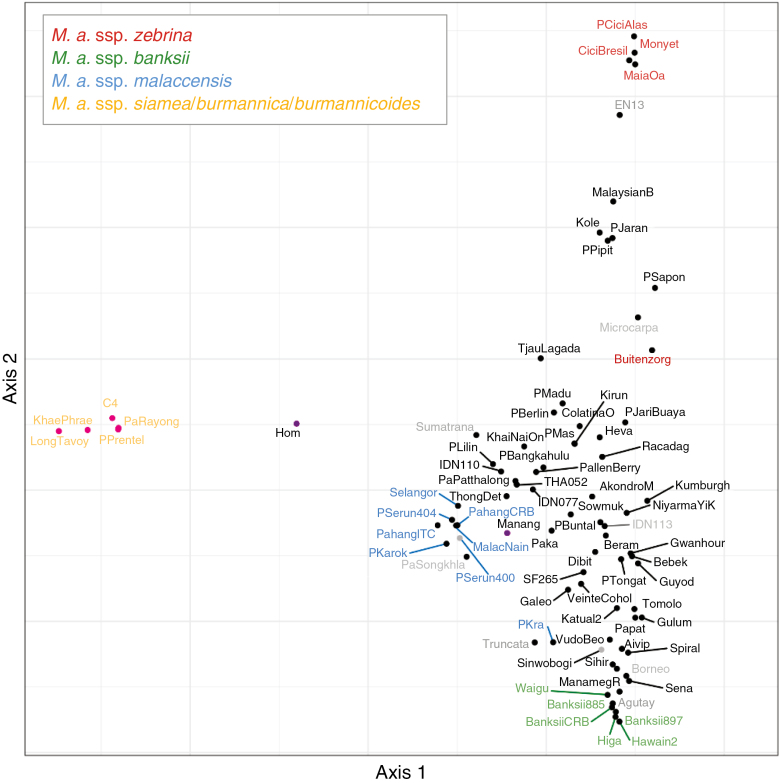
Factorial analysis performed on 32 wild *M. acuminata* accessions with projections of 51 cultivated accessions. The dissimilarity matrix was based on SNPs from WGS data. Pink dots indicate accessions homozygous for chromosomes 2T8 and 8T2 and carrying at least one version of chromosomes 1T9 and 9T1. Black dots indicate accessions homozygous for chromosomes 2 and 8. Purple dots represent heterozygous accessions for the 2/8 translocation. Grey dots represent accessions with an undetermined structure for the 2/8 translocation. Wild accession names are coloured according to their subspecies: yellow, *M. acuminata* ssp. *burmannica, burmannicoides* and *siamea*; green, *M. acuminata* ssp*. banksii*; blue, *M. acuminata* ssp. *malaccensis*; red, *M. acuminata* ssp*. zebrina*; dark grey, other wild *M. acuminata* subspecies. Eigenvalues: axis 1 = 27.27; axis 2 = 21.11.

A factorial analysis was performed on 3092 SNPs identified from Illumina sequences of the 83 diploid wild and cultivated *M. acuminata* accessions (Fig. 5). The first axis separated ssp. *burmannica*, *burmannicoides* and *siamea* accessions from the others, and explained 27 % of the SNP variance. The second axis separated ssp. *zebrina* and *banksii*, and explained 21% of the data variance. The third axis explained an additional 16 % of the data variance and separated ssp. *malaccensis* from the other accessions (Supplementary data Fig.S4).

The five wild accessions displaying only chromosomes 2T8 and 8T2 were confined in one genetic group, i.e. the *burmannica* group, which includes accessions for ssp. *burmannica*, *burmannicoides* and *siamea*. Those accessions were also those in which the translocation between chromosomes 1 and 9 was detected. Heterozygous accessions for chromosomes 2, 8, 2T8 and 8T2 (‘Hom’ and ‘Manang’) corresponded to cultivars and were located between the *burmannica* group and the rest of the accessions. ‘Hom’ was equidistant and isolated between both groups, while ‘Manang’ was closer to the non-*burmannica* group.

## DISCUSSION

We identified two reciprocal translocations in the *Musa acuminata* ssp. *burmannicoides* accession ‘Calcutta 4’ compared with the reference genome sequence, i.e. *M. acuminata* ssp. *malaccensis* ‘DH-Pahang’. One reciprocal translocation involved chromosomes 2 and 8, resulting in chromosomes 2T8 and 8T2. The other one involved chromosomes 1 and 9, resulting in chromosomes 1T9 and 9T1. ‘Calcutta 4’ was found to be homozygous for both translocations.

These translocated chromosome structures were first searched in 13 accessions using 5 kb mate-pair discordant read analysis. In addition, SSJs specific to the distinct chromosome structures were searched in 123 accessions representative of *Musa* diversity involved in cultivated banana for which paired-end sequence data were available.

We found that these methods, particularly SSJ detection, were sensitive to the presence of polymorphism (SNPs and INDELs) and repeated sequences. Thus, only a positive result for the presence of an SSJ could be directly interpreted with confidence. However, in diploid accessions, we expected that an accession in which we detected chromosomes 1T9, 2T8, 2 or 1 also had the complementary chromosome structures 9T1, 8T2, 8 or 9, respectively. Otherwise, this accession would miss a chromosome segment, i.e. a configuration that would most probably be lethal (particularly during the haploid gametic stage). This assumption was confirmed in all cases where it could be verified. This was the case for ‘Pisang Madu’, ‘Manang’ and ‘Malaccensis Nain’ accessions for which chromosome 8 was detected by discordant 5 kb mate-pair read analysis, while not or partially being detected with SSJs.

Among the 36 wild accessions analysed, only five accessions were identified as having the 2T8 and 8T2 structure, most probably in the homozygous state. The same five accessions were the only ones identified as having at least one copy of the 1T9 and 9T1 chromosome structure. Chromosome 9T1 could not be detected in four of them. However, considering that the absence of the complementary structure would probably be lethal, these accessions were assumed to bear the 9T1 chromosomal structure. One of these accessions, ‘Calcutta 4’, proved to be homozygous for both translocations based on segregation analysis and on 5 kb mate-pair discordant read analysis. These five accessions corresponded to all wild accessions tested from ssp. *burmannicoides* (‘Calcutta 4’), *siamea* (‘Pa Rayong’ and ‘Khae Phrae’) and *burmannica* (‘Long Tavoy’ and ‘Pisang Prentel’). Twenty-six wild *M. acuminata* accessions were identified as having the 2 and 8 chromosomal structures, most probably in the homozygous state. Among them, four accessions (‘Malaccensis Nain’, ‘Pahang’, ‘Borneo’ and ‘Maia’Oa’) were shown to be homozygous for these structures based on 5 kb mate-pair discordant read analysis. One last wild *M. acuminata* accession (‘Pisang Serun 400’) could not be structurally characterized in our study.

*Musa balbisiana* and *M. schizocarpa* were only partially characterized using SSJ analysis, but they were previously reported as being homozygous for reference chromosomes 2 and 8 at the 2/8 breakpoint and for chromosomes 1 and 9 at the 1/9 breakpoint (Belser *et al.*, 2018; Baurens *et al.*, 2019).

Among the 87 cultivated accessions tested, only two accessions (‘Manang’ and ‘Hom’) were identified as having the 2/8 translocated structure, both in the heterozygous state. The 1/9 translocated structure was not detected in any cultivars. A total of 84 cultivated accessions were identified as having the 2 and 8 chromosome structure, most probably in the homozygous state. Among them, seven accessions (‘Akondro Mainty’, ‘Pisang Madu’, ‘Galeo’, ‘Pisang Lilin’, ‘Paka’, ‘Grande Naine’ and ‘IDN 110’) proved to be homozygous for these structures based on 5 kb mate-pair discordant read analysis. No structural characterization could be obtained for the diploid cultivated accession (‘Sinwobogi’).

Based on chromosomal pairing irregularities at meiosis in intersubspecific hybrids, Shepherd (1999) suggested the existence of seven *M. acuminata* translocation groups in which accessions were structurally homogeneous. The ‘Standard’ (ST) group is the largest one, consisting of ssp. *banksii*, *microcarpa* and *malaccensis* accessions. The other groups were named according to the geographic origins of their representatives. The ‘Northern Malayan’ (NM) group includes some *malaccensis* accessions, the ‘Northern 1’ group includes some *burmannicoides* and *siamea* accessions, the ‘Northern 2’ group includes other *burmannica* and *siamea* accessions, the ‘Malayan Highland’ group is based on one *truncata* accession and the Javanese group is based on two *zebrina* accessions, whereas the ‘East African’ group is based on one unclassified accession. Martin *et al.* (2017) identified a reciprocal translocation involving chromosomes 1 and 4, compared with the reference genome sequence, in several *malaccensis* accessions, and suggested that it corresponds to the NM group, while the reference genome sequence corresponds to the ST group. The ‘Calcutta 4’ accession considered in this study was used by Shepherd (1999) as a representative of the ‘Northern 1’ group. The ‘Northern 1’ group was suggested to differ from the ST group by two translocations. Our results showed that the two reciprocal translocations between chromosomes 2 and 8 and between chromosomes 1 and 9 corresponded to this ‘Northern 1’ group defined by Shepherd (1999).

*Burmannica*, *siamea* and *burmannicoides* subspecies were originally distinguished based on morphological characters and the geographical distribution (Simmonds, 1962; Perrier *et al.*, 2009). Recent diversity studies based on diversity array technology (DArT) and simple sequence repeat (SSR) markers did not support this separation in three distinct subspecies (Sardos *et al.*, 2016; Christelová *et al.*, 2017). The presence of 1/9 and 2/8 translocations in the *burmannica*, *siamea* and *burmannicoides* accessions we analysed supported the idea of a unified *burmannica* genetic group. However, the homogeneity of this genetic group should be reassessed once the hypothesis of a third translocation specific to the ‘Northern 2’ group, which is assumed to include *burmannica* and *siamea* accessions (Shepherd, 1999), is confirmed. Based on our sampling, the results obtained in this study suggested that translocations 1/9 and 2/8 emerged in the *burmannica* group and supported the hypothesis that the *Musa* reference genome obtained with the *malaccensis* accession ‘DH-Pahang’ represented the ST group of Shepherd (1999) and the ancestral structure.

The *burmannica* genetic group has been shown to constitute an interesting genetic reservoir of disease resistance: ‘Calcutta 4’ shows resistance to various pests and diseases such as nematodes or black leaf streak disease, while ‘Long Tavoy’ and ‘Khae Phrae’ show resistance to *Fusarium oxysporum* f.sp. *cubense* race 4 and nematodes, respectively (Vuylsteke *et al.*, 1992; Jones, 2000; Quénéhervé *et al.*, 2009).

Our study detected only two cultivated accessions (‘Manang’ and ‘Hom’) among the 87 tested as being heterozygous for the 2/8 translocated structure, while none was detected for the 1/9 translocated structure. This supports the hypothesis that the *burmannica* group has not been an important contributor to present banana cultivars (Carreel *et al.*, 1994).

Large chromosomal translocations have been shown to generate segregation distortion and reduced recombination in progeny from structurally heterozygous parents in several plants (Tadmor *et al.*, 1987; Quillet *et al.*, 1995; Jáuregui *et al.*, 2001) including banana (Martin *et al.*, 2017; Baurens *et al*., 2019). Their impact on chromosome segregation is expected to vary depending on the structure of the chromosomes involved (metacentric, acrocentric, etc) and the characteristic of the translocated segments (size, position, gene content, etc.). Characterizing the impact of the two translocations we described on chromosome segregation will be essential to propose breeding strategies to enhance the use of the disease resistance-rich *burmannica* genetic group in breeding programmes.

### Availability of supporting data

Raw sequence reads for the ‘Calcutta 4’ selfing population were deposited in the SRA of the NCBI (BioProject: PRJNA532826). The ‘Calcutta 4’ selfing population genotyping matrix, the breakpoint regions of the ‘Calcutta 4’ assembly, the ‘Maia’Oa’ and ‘Banksii’ long reads including the breakpoint regions of chromosome 8, the Illumina 5 kb reads mapping in the breakpoint regions of chromosomes 1, 2, 8 and 9 of 123 *Musa* accessions, and the VCF file comprising 3092 SNPs for 83 diploid wild and cultivated *Musa acuminata* accessions are available in the download section of the Banana Genome Hub (http://banana-genome-hub.southgreen.fr/download) under AF-C4_genotypingmatrix.tab, Calcutta4_breakpoints.fasta, corrected_long_reads_chr08breakpoint_MaiaOaBanksii.fasta, breakpoints_123Musa.vcf and 2-8_1-9_translocs.vcf names, respectively.

## SUPPLEMENTARY DATA

Supplementary data are available online at https://academic.oup.com/aob and consist of the following. Figure S1: principle of signature segment junction detection. Figure S2: genetic marker statistics along the 11 *Musa acuminata* chromosomes. Figure S3: paired read mapping of 14 *Musa* accessions focused on the two reciprocal translocations detected in ‘Calcutta 4’. Figure S4: factorial analysis performed on 32 wild *M. acuminata* accessions with projection of 51 cultivated accessions. Table S1: accessions tested for their structure on chromosomes 2, 8, 1 and 9. Table S2: genomic co-ordinates of compared sequences. Table S3: genomic position of the SSJs for the translocation 2/8. Table S4: genomic position of the SSJs for the translocation 1/9.

mcz078_suppl_Supplementary_Material

mcz078_suppl_Supplementary_Figure_S1

mcz078_suppl_Supplementary_Figure_S2

mcz078_suppl_Supplementary_Figure_S3

mcz078_suppl_Supplementary_Figure_S4

mcz078_suppl_Supplementary_Table_S1

mcz078_suppl_Supplementary_Table_S2

mcz078_suppl_Supplementary_Table_S3

mcz078_suppl_Supplementary_Table_S4

## FUNDING

This work was supported by the Agropolis Fondation ‘GenomeHarvest project’ (ID 1504-006) through the ‘Investissements d’Avenir’ programme (Labex Agro:ANR-10-LABX-0001-01), the French Government’s ‘Investissements d’Avenir’ FRANCE GENOMIQUE programme (ANR-10-INBS-09) and the CGIAR Research Programme on Roots, Tubers and Bananas (RTB).
