# Supplementary material for: Two large reciprocal translocations characterized in the disease resistance-rich burmannica genetic group of Musa acuminata
Source: Ann Bot. 2019 Jun 26;124(2):319–29. doi: 10.1093/aob/mcz078 (PMC6758587; doi:10.1093/aob/mcz078)
Supplement: mcz078_suppl_Supplementary_Material [file mcz078_suppl_supplementary_material.docx]

## Supplementary figure 1 - Principle of signature segment junction detection

**(A) Signature segment junction definition.** Considering an accession with a standard chromosome structure (in blue, chr 2 and 3) and an accession with an alternative structure, a reciprocal translocation between chromosomes 2 and 3 result in chr 2T3 and 3T2 (in green). All segments (a,b,c,d) are present in both accessions, but some segment combinations and thus segment junctions are specific to chr 2 (a-b), chr 3(c-d), chr 2T3 (a-d) an chr 3T2 (c-d). They are referred to as specific segment junctions (SSJs).

**(B) Accession characterization through paired-end read alignment.** Paired-end reads of accessions to be structurally characterized are aligned on the sequence of the accession serving as reference for the standard structure chr 2 and chr 3 and on the sequence of the accession serving as reference for the alternative structures 2T3 and 3T2. When paired reads overlapped a SSJ’s position (bold), this indicates that the analysed accession bears the same chromosome structure as the reference accession at this SSJ’s position.

## Supplementary figure 2 - Genetic marker statistics along the 11 *Musa acuminata* chromosomes.

**(A-B)** **Representation of marker linkage in ‘Calcutta 4’ self-progeny along *Musa acuminata* reference chromosomes.** Each dot represents linkage between two markers, with the intensity ranging from blue to dark red. **(A)** Markers are plotted according to their physical distance along the *Musa acuminata* reference genome. Boxes along the axes symbolize scaffolds. A linkage break visible at the beginning of reference chromosome 4 most likely resulted from an absence of segregating markers (see Supplementary Fig. 1C) due to the presence of a homozygous region in ‘Calcutta 4’. **(B)** Markers are plotted consecutively.

**(C)** Black and white boxes symbolize scaffolds and their orientation along the pseudo-molecules. Genetic marker and gene densities are represented in orange and blue, respectively, based on a 100 kb window size.

## Supplementary figure 3 - Paired read mapping of 14 *Musa* accessions focused on the two reciprocal translocations detected in ‘Calcutta 4’.

Circos representation of significant discordant read clusters from 14 accessions compared to the *M. acuminata* reference sequence assembly with a focus on paired read clusters detected in the targeted regions of chromosomes 2 and 8 (**A**) and chromosomes 1 and 9 (**B**). Grey internal lines correspond to paired reads with a correct orientation and insert size, red and blue lines correspond to discordant pairs with a higher insert size and reverse mapping orientation, respectively.

## Supplementary figure 4 - Factorial analysis performed on 32 wild *M. acuminata* accessions with projection of 51 cultivated accessions.

The dissimilarity matrix was based on SNPs from WGS data. Pink dots indicate accessions homozygous for chromosomes 2T8 and 8T2 and carrying at least one version of chromosomes 1T9 and 9T1 (see text). Black dots indicate accessions homozygous for chromosomes 2 and 8. Purple dots represent heterozygous accessions for the 2/8 translocation. Grey dots represent accessions with undetermined structure for the 2/8 translocation. Wild accession names are colored according to their subspecies: yellow: *M. a.* ssp. *burmannica, burmannicoides, siamea*; green: *M. a.* ssp. *banksii*; Blue: *M. a.* ssp. *malaccensis;* Red:  *M. a.* ssp. *Zebrina*; Purple: *M. a.* ssp. *Microcarpa*; Dark grey: other wild *M. a.* subspecies. **A**: axis 1/3, **B**: axis 2/3. Eigenvalues: axis 1 = 27.27; axis 2 = 21.11; axis 3 = 15.93.

## Supplementary table 1 - Accessions tested for their structure on chromosomes 2, 8, 1 and 9.

^1^ Baurens et al., 2018, Belser et al., 2018

^2^ Accession ‘PA Songkhla’ is either classified as *M. a.* ssp. *siamea* or unclassified in the Musa Germplasm Information System (<https://www.crop-diversity.org/mgis/>). Literature (Carreel, 1994, Grapin et al., 1998, Sardos et al., 2016) and the diversity factorial analysis detailed in this study do not support the *siamea* classification. It was thus indicated as *M. a.* ssp. with no further detail.

**Supplementary Table 2 - Genomic coordinates of compared sequences.**

**Supplementary Table 3 - Genomic position of the SSJs for the translocation 2/8.**

**Supplementary Table 4 - Genomic position of the SSJs for the translocation 1/9.**
