# Supplementary material for: Two large reciprocal translocations characterized in the disease resistance-rich burmannica genetic group of Musa acuminata
Source: Ann Bot. 2019 Jun 26;124(2):319–29. doi: 10.1093/aob/mcz078 (PMC6758587; doi:10.1093/aob/mcz078)
Supplement: mcz078_suppl_Supplementary_Figure_S1 [file mcz078_suppl_supplementary_figure_s1.pptx]

## Slide 1
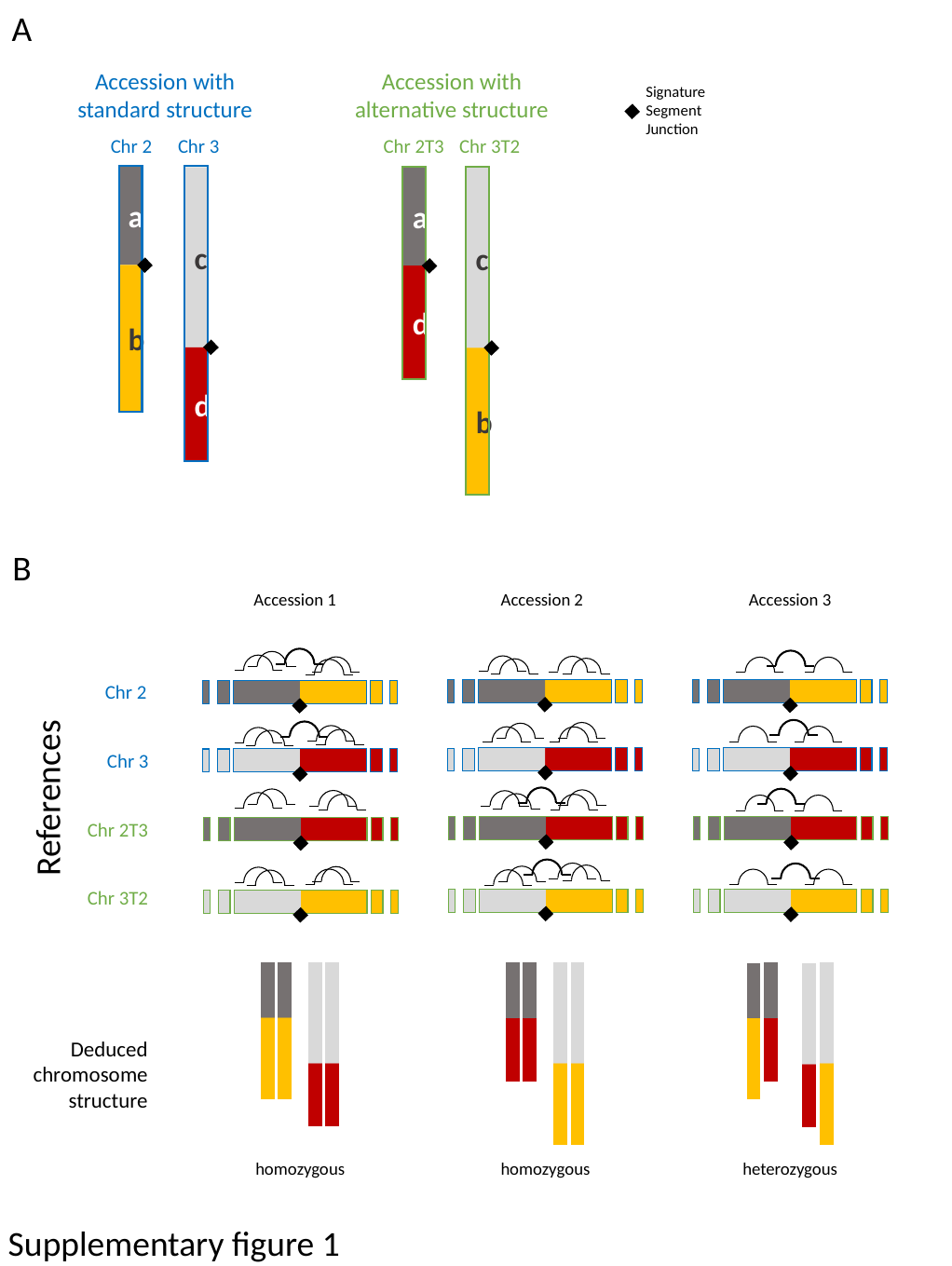

A
Accession with
standard structure
Accession with
alternative structure
Signature Segment Junction
Chr 2
Chr 3
a
c
b
d
Chr 3T2
Chr 2T3
c
a
d
b
B
Accession 1
Accession 2
Accession 3
Chr 2
Chr 3
Chr 2T3
Chr 3T2
References
homozygous
homozygous
heterozygous
Deduced chromosome structure
Supplementary figure 1
