# Supplementary material for: Two large reciprocal translocations characterized in the disease resistance-rich burmannica genetic group of Musa acuminata
Source: Ann Bot. 2019 Jun 26;124(2):319–29. doi: 10.1093/aob/mcz078 (PMC6758587; doi:10.1093/aob/mcz078)
Supplement: mcz078_suppl_Supplementary_Figure_S2 [file mcz078_suppl_supplementary_figure_s2.pptx]

## Slide 1
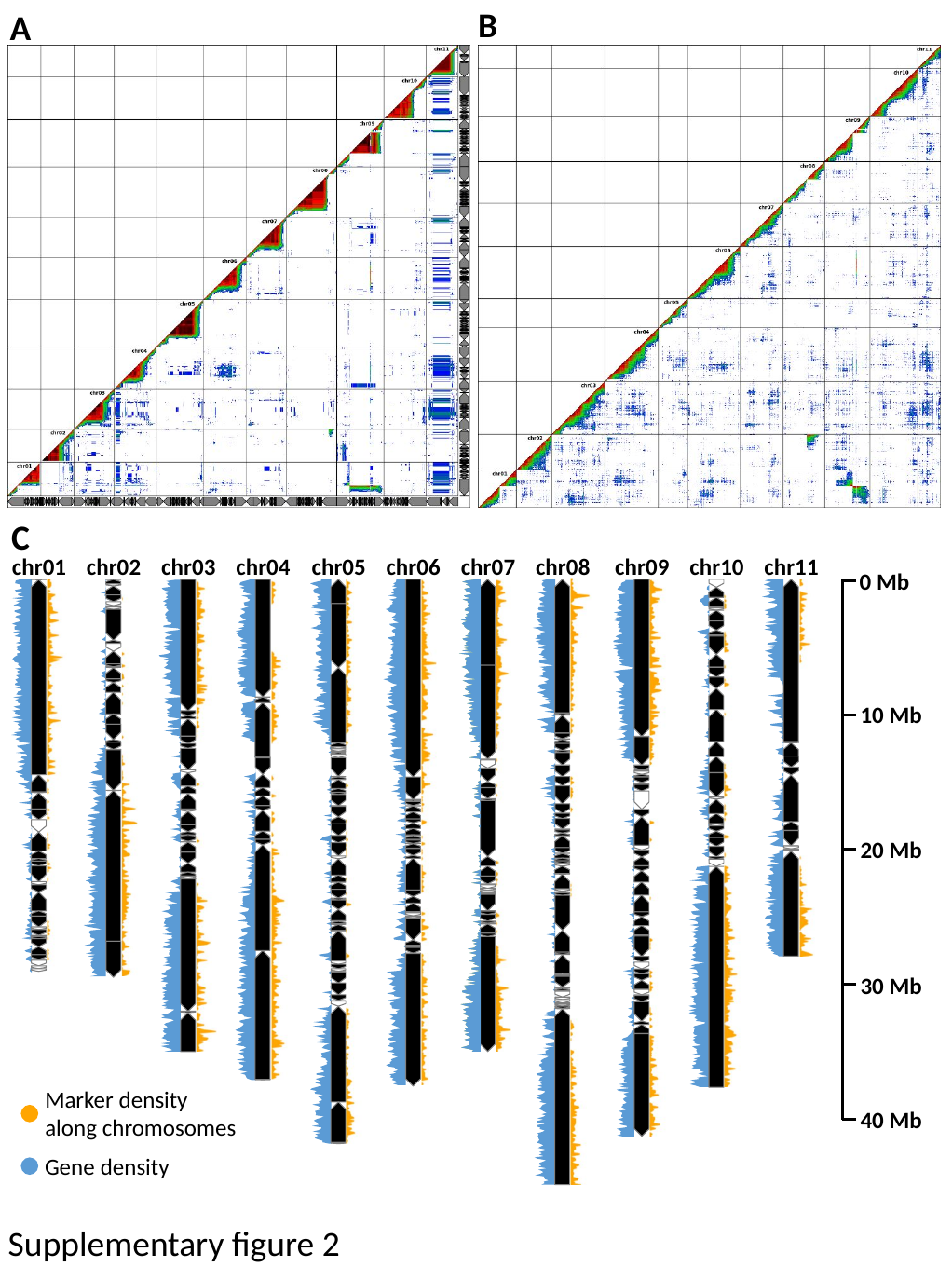

B
A
C
chr09
chr10
chr11
chr05
chr06
chr07
chr08
chr01
chr02
chr03
chr04
0 Mb
10 Mb
20 Mb
30 Mb
40 Mb
Marker density
along chromosomes
Gene density
Supplementary figure 2
