# Supplementary material for: Two large reciprocal translocations characterized in the disease resistance-rich burmannica genetic group of Musa acuminata
Source: Ann Bot. 2019 Jun 26;124(2):319–29. doi: 10.1093/aob/mcz078 (PMC6758587; doi:10.1093/aob/mcz078)
Supplement: mcz078_suppl_Supplementary_Figure_S3 [file mcz078_suppl_supplementary_figure_s3.pptx]

## Slide 1
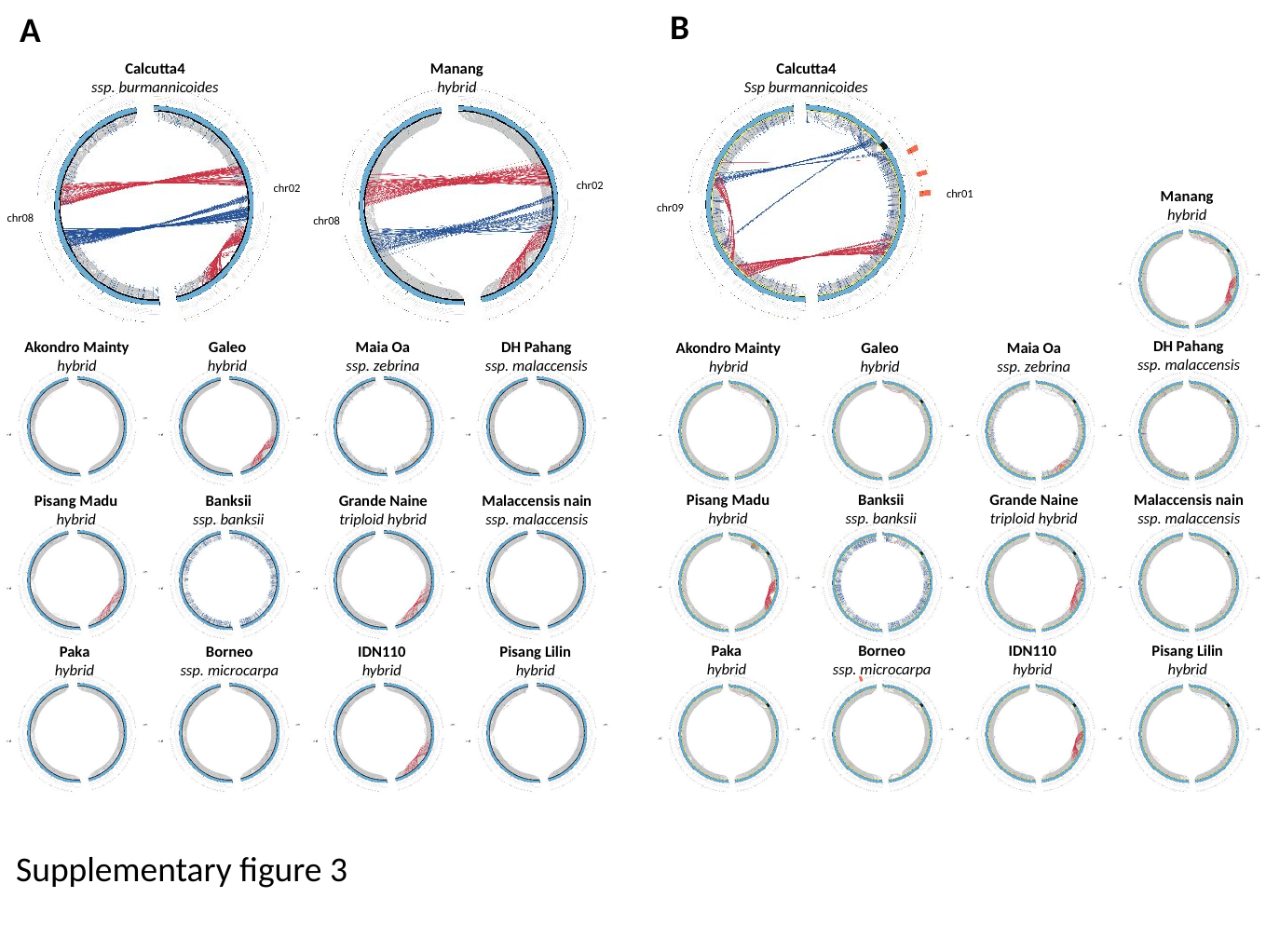

B
A
Calcutta4
Ssp burmannicoides
Manang
hybrid
DH Pahang
ssp. malaccensis
Akondro Mainty
hybrid
Galeo
hybrid
Maia Oa
ssp. zebrina
Malaccensis nain
ssp. malaccensis
Banksii
ssp. banksii
Grande Naine
triploid hybrid
Pisang Madu
hybrid
Borneo
ssp. microcarpa
IDN110
hybrid
Paka
hybrid
Pisang Lilin
hybrid
Calcutta4
ssp. burmannicoides
Manang
hybrid
Akondro Mainty
hybrid
Galeo
hybrid
Maia Oa
ssp. zebrina
DH Pahang
ssp. malaccensis
Pisang Madu
hybrid
Banksii
ssp. banksii
Grande Naine
triploid hybrid
Malaccensis nain
ssp. malaccensis
Paka
hybrid
Borneo
ssp. microcarpa
IDN110
hybrid
Pisang Lilin
hybrid
chr02
chr02
chr01
chr09
chr08
chr08
Supplementary figure 3
