# Supplementary figures and images for: Two large reciprocal translocations characterized in the disease resistance-rich burmannica genetic group of Musa acuminata
Source: Ann Bot. 2019 Jun 26;124(2):319–29. doi: 10.1093/aob/mcz078 (PMC6758587; doi:10.1093/aob/mcz078)

## Slide 1
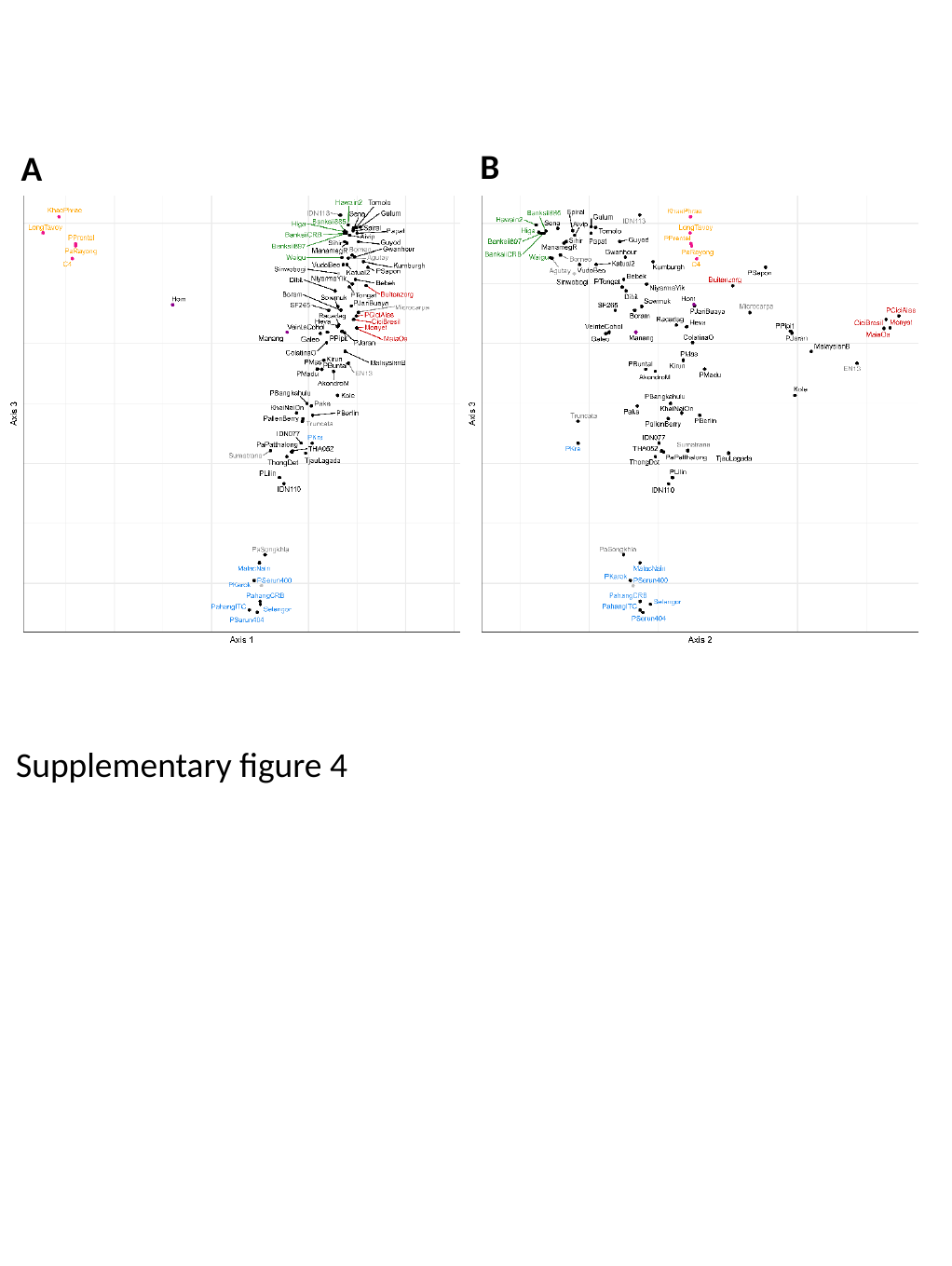

B
A
Supplementary figure 4

Supplement: mcz078_suppl_Supplementary_Figure_S4 [file mcz078_suppl_supplementary_figure_s4.pptx]
