# Supplementary material for: Two large reciprocal translocations characterized in the disease resistance-rich burmannica genetic group of Musa acuminata
Source: Ann Bot. 2019 Jun 26;124(2):319–29. doi: 10.1093/aob/mcz078 (PMC6758587; doi:10.1093/aob/mcz078)
Supplement: mcz078_suppl_Supplementary_Table_S1 [file mcz078_suppl_supplementary_table_s1.docx]

| **Name** | ***group/species*** | **Chromosome 2 and 8 structures** | | | | | | | | | | | | | | | | **Chromosome 1 and 9 structures** | | | | | | |
| --- | --- | --- | --- | --- | --- | --- | --- | --- | --- | --- | --- | --- | --- | --- | --- | --- | --- | --- | --- | --- | --- | --- | --- | --- |
|  |  | **Mate-pair sequencing** | | | **Signature Segment Junctions** | | | | | | | | | | | | **Deduced structure** | **Mate-pair sequencing** | | | **Signature  Segment  Junctions** | | | **Deduced structure** |
|  |  | **Homozygous 02/08** | **Heterozygous** | **Homorygous 2T8/8T2** | **Pahang HD** | | | | **Banksii** | | **Maia'Oa** | | **Calcutta 4** | | | |  | **Homozygous 01/09** | **Heterozygous** | **Homorygous 1T9/9T1** | **Calcutta 4** | | |  |
|  |  |  |  |  | **Ch2** | | **Ch8** | | **Ch8** | | **Ch8** | | **Ch2T8** | | **Ch8T2** | |  |  |  |  | **Chr1T9** | | **Chr9T1** |  |
|  |  |  |  |  | **a-b** | **b-c** | **d-e** | **e-f** | **d-e** | **e-f** | **d-e** | **e-f** | **a-x** | **x-f** | **d-y** | **y-c** |  |  |  |  | **k1-k3** | **k3-h** | **h-k1** |  |
| Banksii (CRB) | *M. a. ssp. banksii* | 1 | 0 | 0 | 115 | 105 |  |  | 126 | 149 |  |  |  |  |  |  | **ST** | 1 | 0 | 0 |  |  |  | **ST** |
| Hawain 2 | *M. a. ssp. banksii* |  |  |  | 148 | 179 |  |  | 125 | 190 |  |  |  |  |  |  | **ST** |  |  |  |  |  |  | **?** |
| M. a. ssp. banksii ITC0885 | *M. a. ssp. banksii* |  |  |  | 128 | 148 | 40 |  | 146 | 180 |  |  |  |  |  |  | **ST** |  |  |  |  |  |  | **?** |
| M. a. ssp. banksii ITCO897 | *M. a. ssp. banksii* |  |  |  | 62 | 99 |  |  | 68 | 117 |  |  |  |  |  |  | **ST** |  |  |  |  |  |  | **?** |
| Waigu | *M. a. ssp. banksii* |  |  |  | 135 | 82 |  |  | 130 | 125 |  |  |  |  |  |  | **ST** |  |  |  |  |  |  | **?** |
| Higa (ITC0464) | *M. a. ssp. banksii* |  |  |  | 115 | 168 |  |  | 145 | 117 |  |  |  |  |  |  | **ST** |  |  |  |  |  |  | **?** |
| Long Tavoy | *M. a. ssp. burmannica* |  |  |  |  |  |  |  |  |  |  |  | 53 | 55 | 99 | 23 | **N1** |  |  |  | 154 |  |  | **N1** |
| Pisang Prentel | *M. a. ssp. burmannica* |  |  |  |  |  |  |  |  |  |  |  | 63 | 69 | 78 | 31 | **N1** |  |  |  | 182 |  |  | **N1** |
| Calcutta 4 | *M. a. ssp. burmannicoïdes* | 0 | 0 | 1 |  |  |  |  |  |  |  |  | 57 | 61 | 63 | 24 | **N1** | 0 | 0 | 1 | 97 |  | 11 | **N1** |
| Agutay (ITC1028) | *M. a. ssp. errans* |  |  |  | 155 | 135 |  |  | 103 | 157 |  |  |  |  |  |  | **ST** |  |  |  |  |  |  | **?** |
| Malaccensis nain | *M. a. ssp. malaccensis* | 1 | 0 | 0 | 103 | 37 |  |  |  |  | 122 |  |  |  |  |  | **ST** | 1 | 0 | 0 |  |  |  | **ST** |
| Pahang | *M. a. ssp. malaccensis* | 1 | 0 | 0 | 87 | 106 | 105 | 132 | 34 |  |  |  |  |  |  |  | **ST** | 1 | 0 | 0 |  |  |  | **ST** |
| Pahang (CRB) | *M. a. ssp. malaccensis* |  |  |  | 67 | 54 | 28 | 27 | 48 | 59 |  |  |  |  |  |  | **ST** |  |  |  |  |  |  | **?** |
| Pisang Karok 391 | *M. a. ssp. malaccensis* |  |  |  | 58 | 129 |  | 18 | 38 | 63 |  |  |  |  |  |  | **ST** |  |  |  |  |  |  | **?** |
| Pisang Kra | *M. a. ssp. malaccensis* |  |  |  | 44 | 90 |  | 19 | 49 | 58 |  |  |  |  |  |  | **ST** |  |  |  |  |  |  | **?** |
| Pisang serun 400 | *M. a. ssp. malaccensis* |  |  |  | 59 |  |  |  |  |  |  |  |  |  |  |  | **?** |  |  |  |  |  |  | **?** |
| Pisang serun 404 | *M. a. ssp. malaccensis* |  |  |  | 76 | 52 |  | 26 | 54 | 69 | 42 | 32 |  |  |  |  | **ST** |  |  |  |  |  |  | **?** |
| Sélangor | *M. a. ssp. malaccensis* |  |  |  | 117 | 80 |  | 28 |  |  | 108 | 65 |  |  |  |  | **ST** |  |  |  |  |  |  | **?** |
| Bornéo | *M. a. ssp. microcarpa* | 1 | 0 | 0 | 80 | 70 |  |  | 74 | 89 |  |  |  |  |  |  | **ST** | 1 | 0 | 0 |  |  |  | **ST** |
| EN 13 IDN075 | *M. a. ssp. microcarpa* |  |  |  | 89 | 97 | 39 | 12 |  |  | 78 | 34 |  |  |  |  | **ST** |  |  |  |  |  |  | **?** |
| Microcarpa | *M. a. ssp. microcarpa* |  |  |  | 93 | 63 |  |  | 48 | 60 | 42 | 51 |  |  |  |  | **ST** |  |  |  |  |  |  | **?** |
| AAs-IDN113 | *M. a. ssp. microcarpa der.* |  |  |  | 56 | 98 |  |  |  |  | 67 | 82 |  |  |  |  | **ST** |  |  |  |  |  |  | **?** |
| Khae Phrae | *M. a. ssp. siamea* |  |  |  |  |  |  |  |  |  |  |  | 74 | 95 | 92 | 19 | **N1** |  |  |  | 142 |  |  | **N1** |
| Pa Rayong | *M. a. ssp. siamea* |  |  |  |  |  |  |  |  |  |  |  | 38 | 37 | 108 | 21 | **N1** |  |  |  | 102 |  |  | **N1** |
| Sumatrana | *M. a. ssp. sumatrana* |  |  |  | 122 | 94 |  | 15 |  |  |  |  |  |  |  |  | **ST** |  |  |  |  |  |  | **?** |
| Truncata | *M. a. ssp. truncata* |  |  |  | 61 | 88 |  | 4 |  |  |  |  |  |  |  |  | **ST** |  |  |  |  |  |  | **?** |
| Buitenzorg | *M. a. ssp. zebrina* |  |  |  | 117 | 100 |  | 23 | 48 | 55 | 54 | 35 |  |  |  |  | **ST** |  |  |  |  |  |  | **?** |
| Cici Brésil | *M. a. ssp. zebrina* |  |  |  | 104 | 83 |  | 21 |  |  | 94 | 96 |  |  |  |  | **ST** |  |  |  |  |  |  | **?** |
| Maia Oa | *M. a. ssp. zebrina* | 1 | 0 | 0 | 120 | 115 |  |  |  |  | 110 | 132 |  |  |  |  | **ST** | 1 | 0 | 0 |  |  |  | **ST** |
| Monyet | *M. a. ssp. zebrina* |  |  |  | 87 | 85 | 56 |  |  |  | 61 | 68 |  |  |  |  | **ST** |  |  |  |  |  |  | **?** |
| Pisang Cici Alas | *M. a. ssp. zebrina* |  |  |  | 71 | 57 |  | 30 |  |  | 84 | 72 |  |  |  |  | **ST** |  |  |  |  |  |  | **?** |
| Pa Songkhla | *M. a. ssp.* ^2^ |  |  |  | 81 | 82 | 110 | 119 | 22 |  |  |  |  |  |  |  | **ST** |  |  |  |  |  |  | **?** |
| Gulum | AAcv |  |  |  | 106 | 125 |  |  | 110 | 137 |  |  |  |  |  |  | **ST** |  |  |  |  |  |  | **?** |
| Guyod | AAcv |  |  |  | 106 | 109 |  |  | 105 | 129 |  |  |  |  |  |  | **ST** |  |  |  |  |  |  | **?** |
| Kole | AAcv |  |  |  | 136 | 136 |  | 36 |  | 67 | 85 | 69 |  |  |  |  | **ST** |  |  |  |  |  |  | **?** |
| Malaysian Blood | AAcv |  |  |  | 122 | 135 | 79 | 37 |  |  | 135 | 158 |  |  |  |  | **ST** |  |  |  |  |  |  | **?** |
| Pa (Patthalong) | AAcv |  |  |  | 59 | 52 |  |  |  |  |  |  |  |  |  |  | **ST** |  |  |  |  |  |  | **?** |
| Pallen Berry | AAcv |  |  |  | 190 | 97 |  | 46 | 93 | 68 |  |  |  |  |  |  | **ST** |  |  |  |  |  |  | **?** |
| Pisang Pipit | AAcv |  |  |  | 73 | 70 | 54 | 30 |  | 25 | 71 | 32 |  |  |  |  | **ST** |  |  |  |  |  |  | **?** |
| Pisang Sapon | AAcv |  |  |  | 74 | 111 | 33 |  | 34 | 43 | 39 | 38 |  |  |  |  | **ST** |  |  |  |  |  |  | **?** |
| Pisang Tongat | AAcv |  |  |  | 84 | 50 |  |  | 93 | 96 |  |  |  |  |  |  | **ST** |  |  |  |  |  |  | **?** |
| Racadag | AAcv |  |  |  | 172 | 128 | 81 |  | 63 | 95 | 80 | 49 |  |  |  |  | **ST** |  |  |  |  |  |  | **?** |
| Sinwobogi | AAcv |  |  |  | 140 |  |  |  | 140 |  |  |  |  |  |  |  | **?** |  |  |  |  |  |  | **?** |
| Tomolo | AAcv |  |  |  | 96 | 95 | 38 |  | 105 | 162 |  |  |  |  |  |  | **ST** |  |  |  |  |  |  | **?** |
| Veinte Cohol | AAcv |  |  |  | 96 | 107 |  |  | 50 | 68 |  | 38 |  |  |  |  | **ST** |  |  |  |  |  |  | **?** |
| Pisang Berlin | AAcv |  |  |  | 95 | 58 |  | 17 | 121 | 106 |  |  |  |  |  |  | **ST** |  |  |  |  |  |  | **?** |
| Colatina Ouro | AAcv |  |  |  | 85 | 74 |  |  | 54 | 55 | 60 | 41 |  |  |  |  | **ST** |  |  |  |  |  |  | **?** |
| Gwanhour | AAcv |  |  |  | 87 | 90 |  |  | 44 | 65 | 36 | 63 |  |  |  |  | **ST** |  |  |  |  |  |  | **?** |
| Niyarma Yik | AAcv |  |  |  | 165 | 160 | 69 |  | 66 | 84 | 79 | 78 |  |  |  |  | **ST** |  |  |  |  |  |  | **?** |
| Sowmuk | AAcv |  |  |  | 119 | 88 | 80 |  | 81 | 86 | 78 | 81 |  |  |  |  | **ST** |  |  |  |  |  |  | **?** |
| Bebek | AAcv |  |  |  | 144 | 87 | 38 |  | 130 | 175 |  |  |  |  |  |  | **ST** |  |  |  |  |  |  | **?** |
| Beram | AAcv |  |  |  | 88 | 75 |  |  | 49 | 80 |  |  |  |  |  |  | **ST** |  |  |  |  |  |  | **?** |
| Heva | AAcv |  |  |  | 125 | 103 | 90 |  |  | 52 | 71 | 65 |  |  |  |  | **ST** |  |  |  |  |  |  | **?** |
| Pisang Jari Buaya | AAcv |  |  |  | 94 | 84 | 61 | 9 | 56 | 48 |  |  |  |  |  |  | **ST** |  |  |  |  |  |  | **?** |
| IDN 077 | AAcv |  |  |  | 61 | 67 | 65 | 54 | 54 | 48 |  |  |  |  |  |  | **ST** |  |  |  |  |  |  | **?** |
| Kirun | AAcv |  |  |  | 98 | 83 |  | 18 | 49 | 71 | 58 |  |  |  |  |  | **ST** |  |  |  |  |  |  | **?** |
| N°110/THA052 | AAcv |  |  |  | 133 | 47 |  |  |  |  | 66 | 46 |  |  |  |  | **ST** |  |  |  |  |  |  | **?** |
| Pisang Jaran | AAcv |  |  |  | 170 | 104 | 104 | 42 |  |  | 153 | 172 |  |  |  |  | **ST** |  |  |  |  |  |  | **?** |
| Pisang Mas | AAcv |  |  |  | 128 | 68 |  |  | 47 | 75 | 67 | 39 |  |  |  |  | **ST** |  |  |  |  |  |  | **?** |
| Dibit | AAcv |  |  |  | 95 | 66 |  |  |  |  | 23 | 67 |  |  |  |  | **ST** |  |  |  |  |  |  | **?** |
| Galeo | AAcv | 1 | 0 | 0 | 111 | 91 |  |  | 117 | 159 |  |  |  |  |  |  | **ST** | 1 | 0 | 0 |  |  |  | **ST** |
| SF 265 | AAcv |  |  |  | 228 | 182 |  |  | 196 | 240 |  |  |  |  |  |  | **ST** |  |  |  |  |  |  | **?** |
| Katual no.2 | AAcv |  |  |  | 88 | 100 |  |  | 96 | 121 |  |  |  |  |  |  | **ST** |  |  |  |  |  |  | **?** |
| Papat | AAcv |  |  |  | 148 | 160 |  |  | 142 | 146 |  |  |  |  |  |  | **ST** |  |  |  |  |  |  | **?** |
| Hom | AAcv |  |  |  | 79 | 29 |  |  | 47 | 66 |  |  |  | 50 | 66 | 12 | **ST+N1** |  |  |  |  |  |  | **?** |
| IDN 110 | AAcv | 1 | 0 | 0 | 67 | 50 | 38 | 12 | 35 | 45 | 42 |  |  |  |  |  | **ST** | 1 | 0 | 0 |  |  |  | **ST** |
| Khai Nai on | AAcv |  |  |  | 100 | 53 |  |  | 68 | 98 |  |  |  |  |  |  | **ST** |  |  |  |  |  |  | **?** |
| Manang | AAcv | 0 | 1 | 0 | 134 | 48 |  |  |  | 103 |  |  | 97 | 49 |  |  | **ST+N1** |  |  |  |  |  |  | **?** |
| Pisang Bangkahulu | AAcv |  |  |  | 75 | 33 |  |  | 40 | 66 |  |  |  |  |  |  | **ST** |  |  |  |  |  |  | **?** |
| Pisang Buntal | AAcv |  |  |  | 160 | 106 |  |  | 48 | 121 |  |  |  | 2 |  |  | **ST** |  |  |  |  |  |  | **?** |
| Thong Det | AAcv |  |  |  | 83 | 47 | 65 | 64 | 77 | 64 |  |  |  |  |  |  | **ST** |  |  |  |  |  |  | **?** |
| Tjau Lagada | AAcv |  |  |  |  | 48 |  |  |  | 79 | 91 |  |  |  |  |  | **ST** |  |  |  |  |  |  | **?** |
| Kumburgh | AAcv |  |  |  | 102 | 102 |  |  | 47 | 61 |  | 33 |  |  |  |  | **ST** |  |  |  |  |  |  | **?** |
| Pisang Madu | AAcv | 1 | 0 | 0 | 118 | 64 |  | 6 |  | 37 |  |  |  |  |  |  | **ST** | 1 | 0 | 0 |  |  |  | **ST** |
| Akondro mainty | AAcv | 1 | 0 | 0 | 92 | 119 |  |  | 96 | 128 |  |  |  |  |  |  | **ST** | 1 | 0 | 0 |  |  |  | **ST** |
| Aivip | AAcv |  |  |  | 76 | 102 |  |  | 130 | 121 |  |  |  |  |  |  | **ST** |  |  |  |  |  |  | **?** |
| Sena | AAcv |  |  |  | 102 |  |  |  | 100 | 149 |  |  |  |  |  |  | **ST** |  |  |  |  |  |  | **?** |
| Spiral | AAcv |  |  |  | 131 | 137 | 49 |  | 138 | 141 |  |  |  |  |  |  | **ST** |  |  |  |  |  |  | **?** |
| Manameg Red | AAcv |  |  |  | 92 | 100 |  |  | 77 | 100 |  |  |  |  |  |  | **ST** |  |  |  |  |  |  | **?** |
| Vudo Beo | AAcv |  |  |  | 149 | 168 |  |  | 86 | 101 |  | 50 |  |  |  |  | **ST** |  |  |  |  |  |  | **?** |
| Sihir | AAcv |  |  |  | 83 | 80 |  |  | 83 | 95 |  |  |  |  |  |  | **ST** |  |  |  |  |  |  | **?** |
| Pisang Lilin | AAcv | 1 | 0 | 0 | 129 | 50 |  | 34 |  | 39 | 69 |  |  |  |  |  | **ST** | 1 | 0 | 0 |  |  |  | **ST** |
| Paka | AAcv | 1 | 0 | 0 | 118 | 79 |  |  | 30 | 48 | 75 |  |  |  |  |  | **ST** | 1 | 0 | 0 |  |  |  | **ST** |
| Pisang Ambon | AAAcv |  |  |  | 69 | 78 |  | 24 | 25 | 51 | 44 | 21 |  |  |  |  | **ST** |  |  |  |  |  |  | **?** |
| Hom (Thong Mokho) | AAAcv |  |  |  | 116 | 97 |  |  | 59 | 136 |  |  |  |  |  |  | **ST** |  |  |  |  |  |  | **?** |
| Grande Naine | AAAcv | 1 | 0 | 0 | 130 | 140 |  |  | 90 | 155 |  |  |  |  |  |  | **ST** | 1 | 0 | 0 |  |  |  | **ST** |
| Koja | AAAcv |  |  |  | 109 | 133 |  | 31 | 49 | 41 | 86 | 43 |  |  |  |  | **ST** |  |  |  |  |  |  | **?** |
| Gros Michel | AAAcv |  |  |  | 160 | 116 |  |  | 135 | 166 |  |  |  |  |  |  | **ST** |  |  |  |  |  |  | **?** |
| Intokatoke | AAAcv |  |  |  | 128 | 154 | 96 | 38 | 61 | 37 | 115 | 133 |  |  |  |  | **ST** |  |  |  |  |  |  | **?** |
| Hom (Sakhon Nakhon) | AAAcv |  |  |  | 100 | 94 | 65 | 49 | 90 | 73 |  |  |  |  |  |  | **ST** |  |  |  |  |  |  | **?** |
| Pisang Kayu | AAAcv |  |  |  | 81 | 102 |  |  | 55 | 95 |  | 31 |  |  |  |  | **ST** |  |  |  |  |  |  | **?** |
| Figue rose | AAAcv |  |  |  | 86 | 55 |  |  | 31 | 73 | 32 | 37 |  |  |  |  | **ST** |  |  |  |  |  |  | **?** |
| Figue rose naine | AAAcv |  |  |  | 85 | 65 |  |  | 29 | 58 | 36 |  |  |  |  |  | **ST** |  |  |  |  |  |  | **?** |
| Pisang Papan | AAAcv |  |  |  | 117 | 71 |  |  | 35 | 36 | 57 | 36 |  |  |  |  | **ST** |  |  |  |  |  |  | **?** |
| Merik | AAAcv |  |  |  | 118 | 114 | 44 |  | 69 | 84 |  | 33 |  |  |  |  | **ST** |  |  |  |  |  |  | **?** |
| Palang | AAAcv |  |  |  | 151 | 144 | 35 |  | 154 | 174 |  |  |  |  |  |  | **ST** |  |  |  |  |  |  | **?** |
| Wh-o-Gu | AAAcv |  |  |  | 88 | 113 |  |  | 14 | 33 | 52 | 66 |  |  |  |  | **ST** |  |  |  |  |  |  | **?** |
| Mnalouki | AABcv |  |  |  | 83 | 61 |  |  | 77 | 104 |  |  |  |  |  |  | **ST** |  |  |  |  |  |  | **?** |
| Luba | AABcv |  |  |  | 81 | 85 |  |  | 31 | 39 |  | 29 |  |  |  |  | **ST** |  |  |  |  |  |  | **?** |
| Laknao | AABcv |  |  |  | 69 | 46 |  |  | 31 | 68 |  |  |  |  |  |  | **ST** |  |  |  |  |  |  | **?** |
| Lady Finger | AABcv |  |  |  | 66 | 44 | 27 | 20 | 64 | 80 |  |  |  |  |  |  | **ST** |  |  |  |  |  |  | **?** |
| Pisang Rajah | AABcv |  |  |  | 72 | 56 |  | 6 | 55 | 69 |  |  |  |  |  |  | **ST** |  |  |  |  |  |  | **?** |
| Pisang Kelat | AABcv |  |  |  | 84 | 59 |  | 3 | 33 | 21 | 18 | 28 |  |  |  |  | **ST** |  |  |  |  |  |  | **?** |
| Mkono Wa Tembo | AABcv |  |  |  | 94 | 75 |  |  | 80 | 78 |  |  |  |  |  |  | **ST** |  |  |  |  |  |  | **?** |
| Prata Ana | AABcv |  |  |  | 158 | 129 |  | 18 | 142 | 148 |  |  |  |  |  |  | **ST** |  |  |  |  |  |  | **?** |
| Maia Maoli | AABcv |  |  |  | 100 | 58 |  |  | 57 | 73 |  |  |  |  |  |  | **ST** |  |  |  |  |  |  | **?** |
| Popoulou CMR | AABcv |  |  |  | 76 | 52 |  |  | 53 | 76 |  |  |  |  |  |  | **ST** |  |  |  |  |  |  | **?** |
| Kingala 1 | AABcv |  |  |  | 105 | 82 |  | 20 | 70 | 77 |  |  |  |  |  |  | **ST** |  |  |  |  |  |  | **?** |
| Poteau Géant | ABBcv |  |  |  | 79 | 27 |  |  | 25 | 39 |  |  |  |  |  |  | **ST** |  |  |  |  |  |  | **?** |
| Monthan | ABBcv |  |  |  | 87 | 38 | 33 |  |  | 33 |  |  |  |  |  |  | **ST** |  |  |  |  |  |  | **?** |
| Ice Cream | ABBcv |  |  |  |  | 41 |  |  | 25 | 39 |  |  |  |  |  |  | **ST** |  |  |  |  |  |  | **?** |
| Pelipita | ABBcv |  |  |  | 105 | 25 |  |  | 74 | 68 |  |  |  |  |  |  | **ST** |  |  |  |  |  |  | **?** |
| Namwa Khom | ABBcv |  |  |  | 108 | 26 |  |  | 42 |  |  |  |  |  |  |  | **ST** |  |  |  |  |  |  | **?** |
| Red Yade | ABBcv |  |  |  | 218 | 18 |  |  | 72 | 85 |  |  |  |  |  |  | **ST** |  |  |  |  |  |  | **?** |
| Saba | ABBcv |  |  |  | 96 | 77 | 17 |  | 63 | 101 |  |  |  |  |  |  | **ST** |  |  |  |  |  |  | **?** |
| Kunnan | ABcv |  |  |  | 58 | 40 |  |  | 27 | 37 |  |  |  |  |  |  | **ST** |  |  |  |  |  |  | **?** |
| Safet Velchi | ABcv |  |  |  | 39 | 47 | 11 |  | 45 | 42 |  |  |  |  |  |  | **ST** |  |  |  |  |  |  | **?** |
| Tonton Kepa | AScv |  |  |  | 127 | 187 |  | 3 | 69 | 69 |  |  |  |  |  |  | **ST** |  |  |  |  |  |  | **?** |
| Wompa | AScv |  |  |  | 135 | 136 | 98 |  |  |  | 29 | 64 |  |  |  |  | **ST** |  |  |  |  |  |  | **?** |
| Butuhan | *Musa balbisiana* |  |  |  | 102 | 12 |  |  |  |  |  |  |  |  |  |  | **ST^1^** |  |  |  |  |  |  | **ST^1^** |
| Pisang Klutuk Wulung | *Musa balbisiana* |  |  |  | 71 | 14 |  |  |  |  |  |  |  |  |  |  | **ST^1^** |  |  |  |  |  |  | **ST^1^** |
| Schizocarpa | *Musa schizocarpa* |  |  |  | 131 | 115 |  |  |  |  |  |  |  |  |  |  | **ST^1^** |  |  |  |  |  |  | **ST^1^** |
| Schizocarpa | *Musa schizocarpa* |  |  |  | 140 | 167 | 78 | 4 |  |  |  |  |  |  |  |  | **ST** |  |  |  |  |  |  | **ST^1^** |
