# Supplementary material for: Two large reciprocal translocations characterized in the disease resistance-rich burmannica genetic group of Musa acuminata
Source: Ann Bot. 2019 Jun 26;124(2):319–29. doi: 10.1093/aob/mcz078 (PMC6758587; doi:10.1093/aob/mcz078)
Supplement: mcz078_suppl_Supplementary_Table_S2 [file mcz078_suppl_supplementary_table_s2.docx]

**Supplementary Table 2 - Genomic coordinates of compared sequences.**

|  | ‘DH-Pahang’ reference genome | ‘Calcutta 4’ assembly |
| --- | --- | --- |
| Translocation 2/8 | chr02:29268836-29274837 | utg154:2411918-2417918 |
|  | chr08:37722146-37728147 | utg170:2941233-2947233 |
| Translocation 1/9 | chr01:8219188-8289189 | utg94:4743017-4813018 |
|  | chr09:11554857-11624857 | utg195:5581660-5651463 |
