# Supplementary material for: Two large reciprocal translocations characterized in the disease resistance-rich burmannica genetic group of Musa acuminata
Source: Ann Bot. 2019 Jun 26;124(2):319–29. doi: 10.1093/aob/mcz078 (PMC6758587; doi:10.1093/aob/mcz078)
Supplement: mcz078_suppl_Supplementary_Table_S3 [file mcz078_suppl_supplementary_table_s3.docx]

**Supplementary Table 3 - Genomic position of the SSJs for the translocation 2/8.**

|  | **Chromosome 2** | | **Chromosome 8** | |
| --- | --- | --- | --- | --- |
| SSJ | **a-b** | **b-c** | **d-e** | **e-f** |
| ‘DH-Pahang’ reference genome | chr02:29271222 | chr02:29272451 | chr08:37723288 | chr08:37727005 |
|  | **Chromosome 2T8** | | **Chromosome 8T2** | |
| SSJ | **a-x** | **x-f** | **d-y** | **y-c** |
| ‘Calcutta 4’ assembly | utg154:2414601 | utg154:2415237 | utg170:2943641 | utg170:2944825 |
