# Supplementary material for: Two large reciprocal translocations characterized in the disease resistance-rich burmannica genetic group of Musa acuminata
Source: Ann Bot. 2019 Jun 26;124(2):319–29. doi: 10.1093/aob/mcz078 (PMC6758587; doi:10.1093/aob/mcz078)
Supplement: mcz078_suppl_Supplementary_Table_S4 [file mcz078_suppl_supplementary_table_s4.docx]

**Supplementary Table 4 - Genomic position of the SSJs for the translocation 1/9.**

|  | **Chromosome 1T9** | | **Chromosome 9T1** |
| --- | --- | --- | --- |
| SSJ | k_1_-k_3_ | k_3_-h | h-k_1_ |
| ‘Calcutta 4’ assembly | utg94:4774156 | utg94:4778354 | utg195:5616816 |
